# p40 homodimers bridge ischemic tissue inflammation and heterologous alloimmunity in mice via IL-15 transpresentation

**DOI:** 10.1172/JCI172760

**Published:** 2024-01-25

**Authors:** Hidetoshi Tsuda, Karen S. Keslar, William M. Baldwin, Peter S. Heeger, Anna Valujskikh, Robert L. Fairchild

**Affiliations:** 1 Department of Inflammation & Immunity, Lerner Research Institute, Cleveland, Ohio, USA.; 2 Transplant Center, Cleveland Clinic, Cleveland, Ohio, USA.; 3 Icahn School of Medicine at Mount Sinai, New York, New York, USA.

**Keywords:** Transplantation, Adaptive immunity, Innate immunity, Organ transplantation

## Abstract

Virus-induced memory T cells often express functional cross-reactivity, or heterologous immunity, to other viruses and to allogeneic MHC molecules that is an important component of pathogenic responses to allogeneic transplants. During immune responses, antigen-reactive naive and central memory T cells proliferate in secondary lymphoid organs to achieve sufficient cell numbers to effectively respond, whereas effector memory T cell proliferation occurs directly within the peripheral inflammatory microenvironment. Mechanisms driving heterologous memory T cell proliferation and effector function expression within peripheral tissues remain poorly understood. Here, we dissected proliferation of heterologous donor-reactive memory CD8^+^ T cells and their effector functions following infiltration into heart allografts with low or high intensities of ischemic inflammation. Proliferation within both ischemic conditions required p40 homodimer–induced IL-15 transpresentation by graft DCs, but expression of effector functions mediating acute allograft injury occurred only in high-ischemic allografts. Transcriptional responses of heterologous donor-reactive memory CD8^+^ T cells were distinct from donor antigen–primed memory CD8^+^ T cells during early activation in allografts and at graft rejection. Overall, the results provide insights into mechanisms driving heterologous effector memory CD8^+^ T cell proliferation and the separation between proliferation and effector function that is dependent on the intensity of inflammation within the tissue microenvironment.

## Abstract 2

Heterolgous memory CD8^+ ^T cells are activated to mediate acute injury in high, but not low, ischemic allografts in mice.

## Graphical abstract

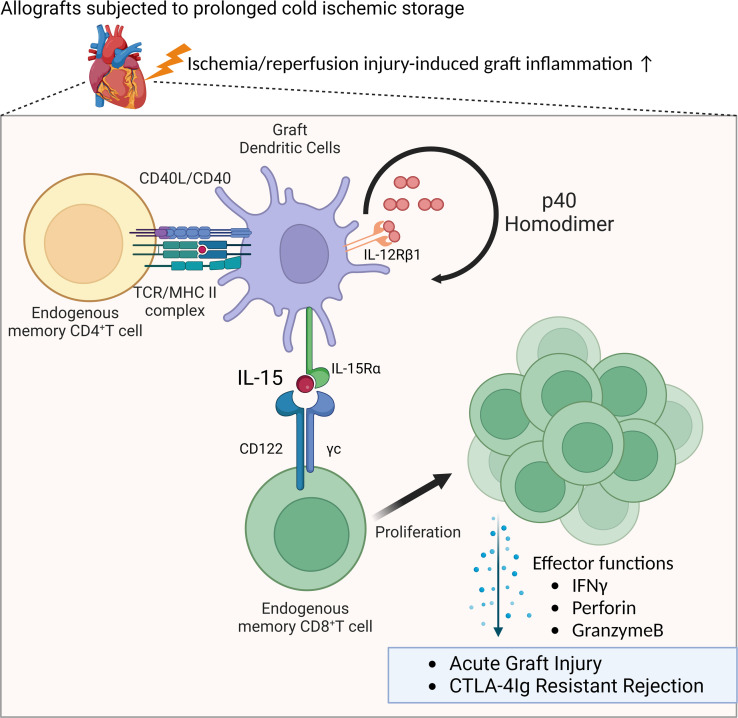

## Introduction

Effective adaptive immune responses require the proliferation and expression of effector function by the limited number of antigen-reactive lymphocyte clones to achieve the numbers needed to mediate the response. For naive and central memory T cells this activation occurs in the secondary lymphoid organs followed by their trafficking to the response site (1–4). In contrast, effector memory T cells responding to an inflammatory stimulus must first infiltrate the peripheral tissue site and then undergo antigen-driven proliferation plus expression of effector function within the inflammatory site. Whereas proliferation of antigen-reactive memory T cells in secondary lymph nodes following challenge with bacteria and viruses is driven by IL-12, IL-15, IL-18, and type I IFN (5–8), cytokine pathways driving effector memory CD8^+^ T cell expansion in tissue sites with challenge antigens remain poorly defined.

Memory T cells generated in response to various microbial pathogens and environmental antigens are often functionally cross-reactive to allogeneic MHC molecules, an example of heterologous immunity. These memory T cells can undermine allograft outcomes in recipients with no prior exposure to the donor alloantigens (9–14). We previously reported that CD4^+^ and CD8^+^ memory T cells with donor reactivity infiltrate vascularized heart allografts within 12–24 hours of graft reperfusion in naive mice housed under specific pathogen–free conditions (15–17). The endogenous donor-reactive memory CD8^+^ T cells are activated within the allografts to express functions, including IFN-γ and granzyme B, that increase the graft inflammation at this early time after transplant. When the transplant is performed after a minimal period of graft cold ischemic storage (CIS), a lower intensity of ischemia/reperfusion injury–induced (IRI-induced) inflammation is generated following vascularization of the graft, and the effector functions expressed by the graft-infiltrating donor-reactive memory CD8^+^ T cells are detectable at low levels (15, 18). In contrast, imposing a clinically relevant period of prolonged CIS on the allografts prior to transplant increases the intensity of IRI-induced graft inflammation with marked increases in heterologous donor-reactive memory CD8^+^ T cell proliferation within the graft. Furthermore, these CD8^+^ T cells are activated to directly mediate acute graft injury and rejection despite recipient conditioning with CTLA-4Ig costimulatory blockade, a treatment that prevents allograft rejection under low-inflammatory conditions (16, 17). Building on our previous work, we herein uncover an unanticipated mechanism that links local p40 homodimer (p40HD) production to drive graft-infiltrating heterologous memory CD8^+^ T cell proliferation via an intragraft, DC-dependent IL-15–driven pathway. Peri-transplant IL-15 inhibition prevents intra-allograft memory T cell expansion and prolongs allograft survival despite the presence of the highly inflammatory microenvironment induced by prolonged CIS.

Beyond its importance in transplantation, our identification of a pathway that promotes antigen-reactive effector T cell proliferation and expression of effector function within the pathogenic allograft microenvironment provides proof of principle that analogous inhibitory approaches could be effective for treating autoimmune diseases, while approaches aimed at boosting local effector memory T cell proliferation and effector function could be an effective strategy for treating malignancies.

## Results

### p40HDs increase IL-15 production within minimally ischemic allografts.

**Figure 1:**
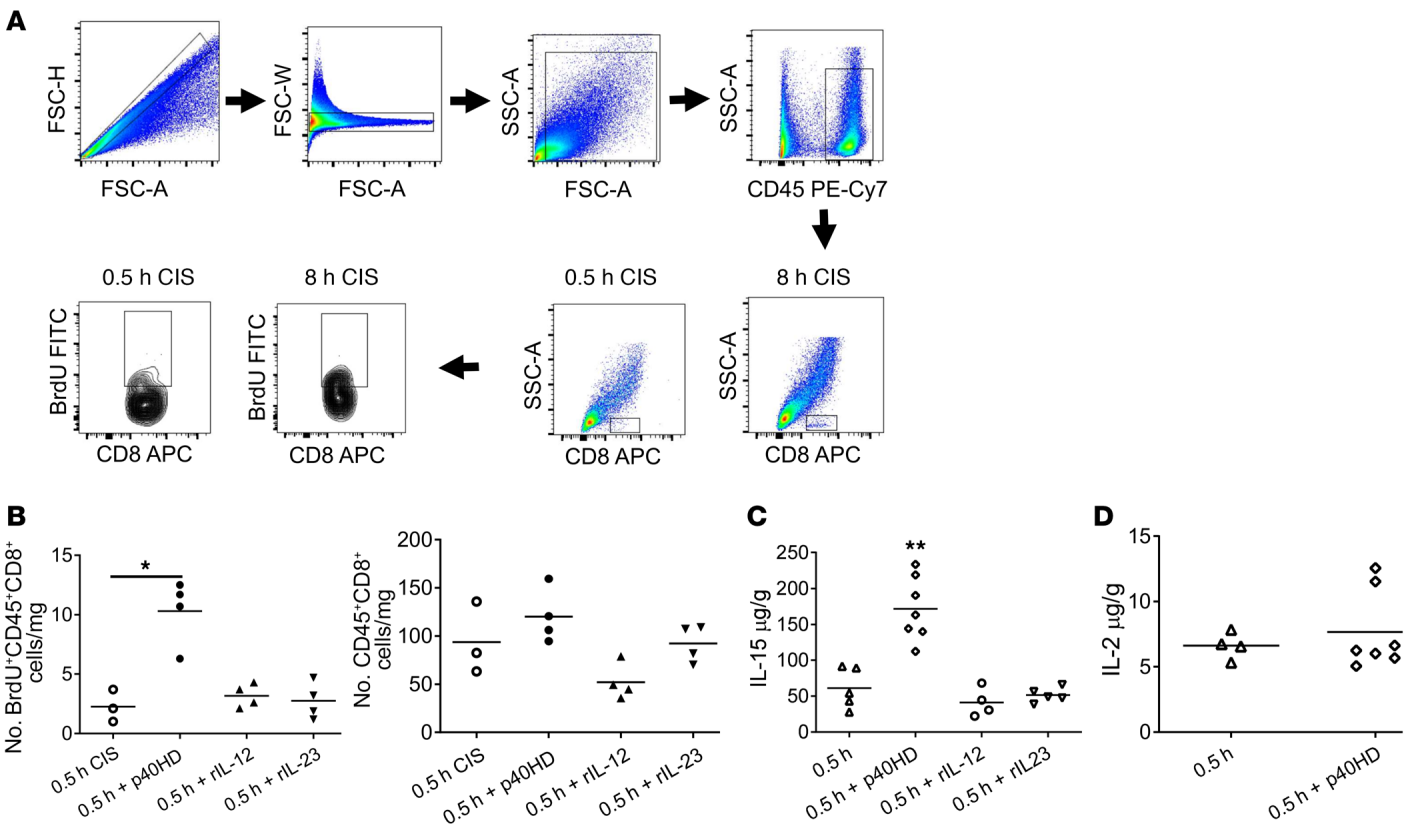
p40HD increases IL-15 production within allografts subjected to 30 minutes of CIS prior to transplant. (**A**) A/J cardiac allografts subjected to either 0.5 or 8 hours of CIS were transplanted into C57BL/6 mice. All recipients were injected with 100 μg BrdU i.p. on days 0 and 1 after transplant. On day 2 after transplant, allografts were harvested and digested, and cell suspensions were stained with antibody and analyzed by flow cytometry using the gating strategy shown to assess the proliferation of graft-infiltrating CD8^+^ T cells in allografts subjected to each of the CIS conditions. (**B**) Groups of C57BL/6 mice (*n* = 3–4 per group) received A/J cardiac allografts subjected to 0.5 hours of CIS. The indicated recipients were treated with either 2 μg recombinant p40HDs, recombinant IL-12, or recombinant IL-23 i.v. on day 1 after transplant. All recipients were injected with 100 μg BrdU i.p. on days 0 and 1 after transplant. The next day, allografts were harvested and digested, and cell suspensions were stained with antibody and analyzed by flow cytometry to assess the proliferation of infiltrating CD8^+^ T cells. *P < 0.05 as determined by the Kruskal-Wallis test. (**C** and **D**) Groups of C57BL/6 (*n* = 4–7 per group) mice received A/J cardiac allografts subjected to 0.5 hours of CIS. The indicated allograft recipients were treated with 2 μg recombinant p40HDs, recombinant IL-12, or recombinant IL-23 i.v. on day 1 after transplant. On day 2 after transplant, grafts were harvested and homogenized, and levels of IL-15 (**C**) and IL-2 (**D**) were tested by ELISA. **P < 0.01 vs. 0.5 hours CIS, as determined by the Kruskal-Wallis or the Mann-Whitney nonparametric test.

Imposition of greater versus minimal CIS time prior to heart allografts provokes greater IRI that includes increased proliferation of graft-infiltrating endogenous memory CD8^+^ T cells (Figure 1A). Our previous work implicated p40HD as a potential driver of endogenous donor-reactive memory CD8^+^ T cell proliferation in the high-ischemic allografts (17) (summarized in Supplemental Figure 1; supplemental material available online with this article; https://doi.org/10.1172/JCI172760DS1). Confirming and extending these previous observations, injection of p40HD, but not IL-12 or IL-23 heterodimers, into recipients of allografts subjected to minimal CIS (low inflammation) provoked a more than 4-fold increase in endogenous memory CD8^+^ T cell proliferation within the allografts (Figure 1B). While p40HD signals through IL-12Rb1 dimers (19), we observed that only about 40% of the CD8^+^ T cells proliferating within allografts expressed IL-12Rb1, regardless of CIS time conferring low or high IRI-induced inflammation in the allograft (Supplemental Figure 2). The greater BrdU incorporation by the IL-12Rb1^–^ CD8^+^ T cells suggested that p40HDs function via an indirect, IL-12Rb1–independent mechanism.

**Figure 2:**
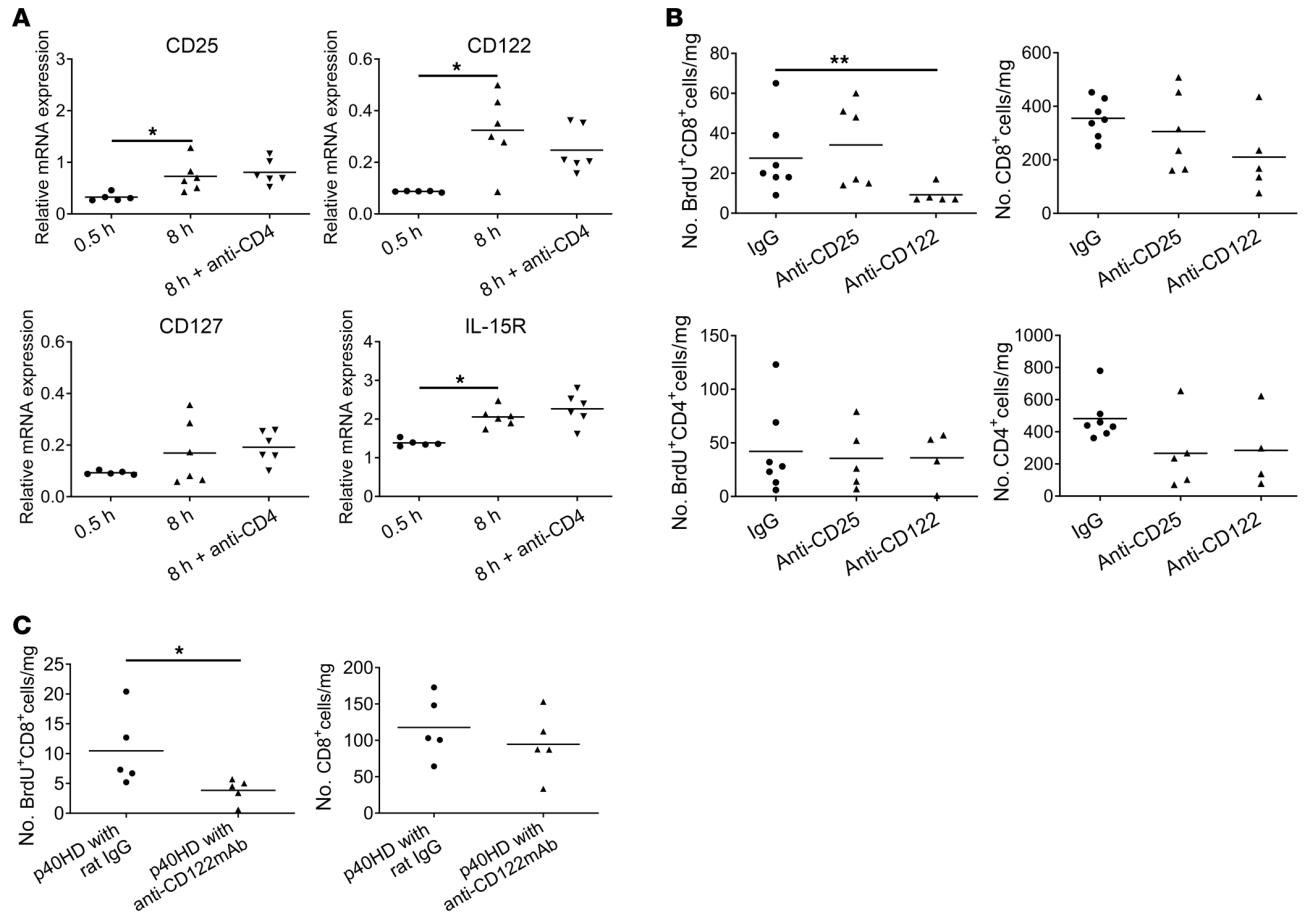
Anti-CD122 mAb but not anti-CD25 mAb inhibits endogenous memory CD8^+^ T cell proliferation within highly ischemic allografts. (**A**) C57BL/6 (*n* = 5–6 per group) mice received A/J cardiac allografts subjected to 0.5 or 8 hours of CIS. Recipients of 8-hour-CIS allografts were treated with 200 μg control rat IgG or anti-CD4 mAb on days –3, –2, and –1 before transplant. Grafts were harvested on day 2, infiltrating CD8^+^ T cells were enriched by negative selection, and total RNA was isolated and tested for expression of the indicated cytokine receptor mRNAs by quantitative PCR. Results shown indicate relative expression by infiltrating CD8^+^ T cells versus expression in purified splenic CD8^+^ T cells from naive A/J mice. *P < 0.05 as determined by Kruskal-Wallis test. (**B**) C57BL/6 mice (*n* = 5–7 per group) received A/J cardiac allografts subjected to 8 hours of CIS and were treated with 100 μg BrdU and either 100 μg control rat IgG, anti-CD25 mAb, or anti-CD122 mAb i.p. on days 0 and 1. On day 2, allografts were harvested and digested, and cell suspensions were stained with antibody and analyzed by flow cytometry to assess total numbers and proliferation of infiltrating memory CD4^+^ and CD8^+^ T cells. **P < 0.01 as determined by the Kruskal-Wallis test. (**C**) C57BL/6 mice (*n* = 5 per group) received A/J cardiac allografts subjected to 0.5 hours of CIS. The indicated recipients were treated with 100 μg BrdU and 200 μg control rat IgG or anti-CD122 mAb i.p. on days 0 and 1 and with 2 μg recombinant p40HDs i.v. on day 1. On day 2, allografts were harvested and digested, and cell suspensions were stained with antibody and analyzed by flow cytometry to assess total numbers and proliferation of infiltrating memory CD8^+^ T cells. *P < 0.05 as determined by Mann-Whitney nonparametric test.

Following injection of p40HD into recipients of low-ischemic allografts, we observed marked increases in IL-15 production in the allograft (Figure 1C) without effects on IL-2 production (Figure 1D). Neither recombinant p40-p35/IL-12 heterodimer nor p40-p19/IL-23 heterodimer stimulated IL-15 production in the low-ischemic allografts, demonstrating the specificity of the p40HD. To test whether p40HD-induced IL-15 drives intra-allograft proliferation of memory CD8^+^ T cells, we first treated recipients of minimally ischemic allografts and p40HD administration with anti-CD122 mAb at transplant and analyzed IL-15R expression and endogenous memory T cell proliferation on day 2 after transplant. These assays showed that the isolated memory CD8^+^ T cells expressed mRNA encoding receptor components for the IL-2 and IL-15 (CD25, CD122, and IL-15Rα) receptors that was increased in memory CD8^+^ T cells isolated from prolonged versus minimal CIS allografts (Figure 2A). In support of these results, flow cytometry analyses of non-proliferating (BrdU^–^) and proliferating (BrdU^+^) CD8^+^ T cells in low- and high-ischemic cardiac allografts indicated significant increases in CD122^+^ proliferating versus non-proliferating CD8^+^ T cells in the high- versus low-ischemic allografts (Supplemental Figure 3, A and B). Expression of CD25 and CD127 was low on proliferating CD8^+^ T cells in the high-ischemic allografts, and there was a slight but non-significant increase in proliferating CD8^+^ T cells expressing IL-15Rα in the high- versus low-ischemic allografts. Furthermore, anti-CD122 mAb markedly decreased memory CD8^+^ T cell proliferation within the high-ischemic allografts, whereas treatment with anti-CD25 (anti–IL-2Rα chain) mAb had no effect (Figure 2B). Neither cytokine receptor blocking antibody affected memory CD4^+^ T cell proliferation within the high-ischemic allografts. Administration of peri-transplant anti-CD122 mAb also inhibited the p40HD-induced increase in memory CD8^+^ T cell proliferation within low-ischemic allografts (Figure 2C), providing a causal link between p40HD-induced proliferation and CD122 expression.

### p40HD-induced IL-15 requires resident DCs in heart allografts.

**Figure 3:**
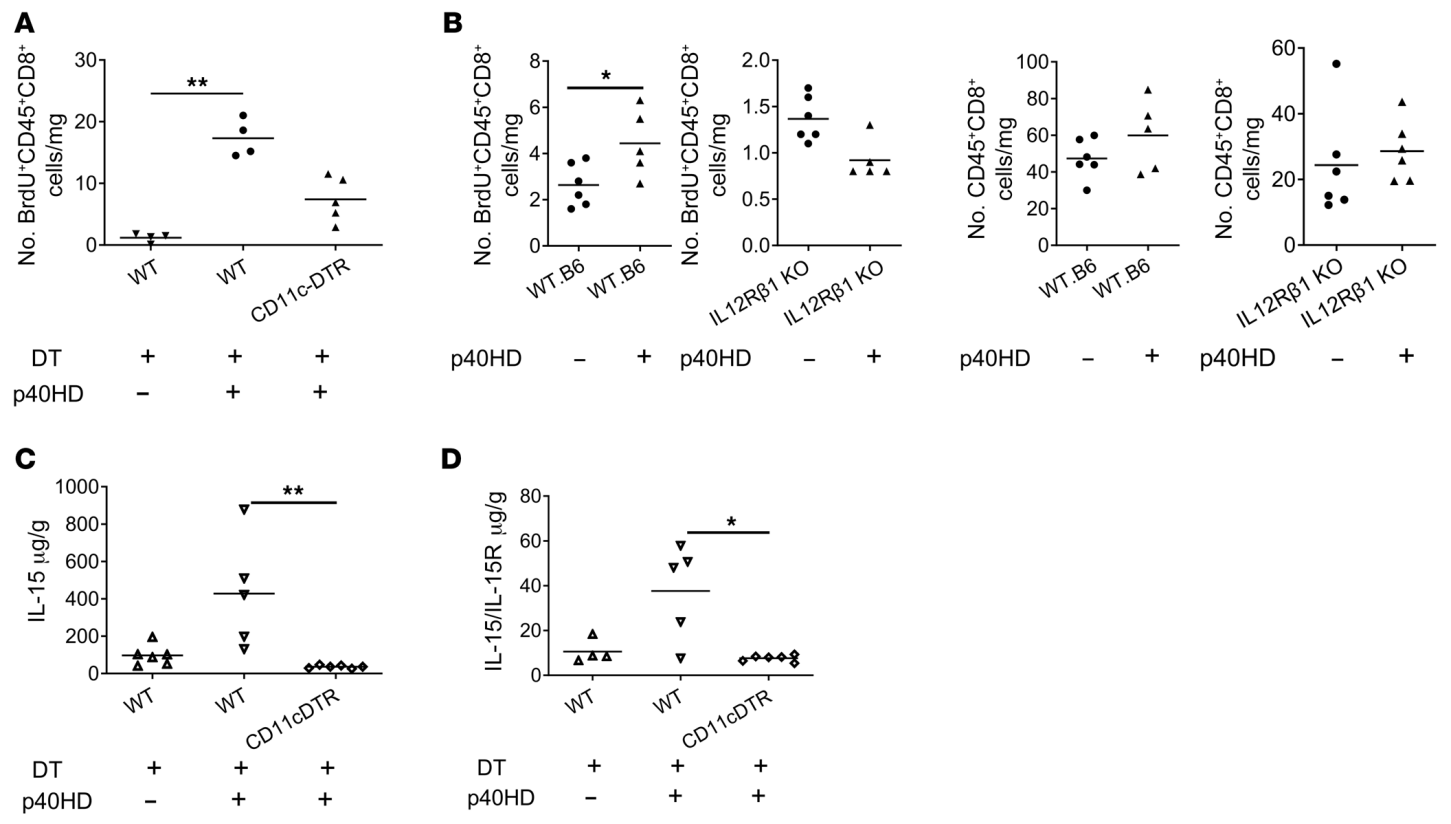
p40HD-induced IL-15 production and endogenous memory CD8^+^ T cell proliferation in allografts subjected to minimal CIS are obviated by graft CD11c^+^ cell depletion. (**A**) Groups of A/J mice (*n* = 4–5 per group) received cardiac allografts subjected to 0.5 hours of CIS from either C57BL/6 or B6.DTR-CD11c transgenic mice that were treated with or without diphtheria toxin (DT; 40 ng/g body weight) on days –2 and –1 before transplantation. On day 1 after transplant, indicated recipients received 2 μg recombinant p40HDs i.v., and all recipients were injected with 100 μg BrdU i.p. on days 0 and 1. The next day, allografts were harvested and digested, and aliquots of single-cell suspensions were stained with antibody and analyzed by flow cytometry to quantitate the proliferation of memory CD8^+^ T cells by BrdU incorporation. **P < 0.01 as determined by the Kruskal-Wallis test. (**B**) Cardiac allografts from wild-type (WT) C57BL/6 or B6.IL12Rβ1^–/–^ mice were subjected to 0.5 hours of CIS and transplanted to groups of BALB/c mice (*n* = 5–6). Recipients were injected with 100 μg BrdU i.p. on days 0 and 1 after transplant and received PBS or 2 μg recombinant p40HDs i.v. on day 1 after transplant. The next day, allografts were harvested and digested, and aliquots of single-cell suspensions were stained with antibody and analyzed by flow cytometry to quantitate the infiltration and the BrdU incorporation of memory CD8^+^ T cells. *P < 0.05 as determined by the Mann-Whitney nonparametric test. (**C** and **D**) On day 2 after transplant, total protein was purified from harvested grafts of indicated recipients, and IL-15 (**C**) or IL-15/IL-15R complex (**D**) levels were tested by ELISA. *P < 0.05, **P < 0.01 as determined by the Kruskal-Wallis test.

To test whether allograft DCs were required for p40HD-induced endogenous memory CD8^+^ T cell proliferation within allografts subjected to minimal CIS, we treated B6.CD11c-DTR and wild-type B6 heart allograft donors with diphtheria toxin (DT) on the day prior to graft harvest and minimal CIS before transplant to complete-MHC-mismatched A/J recipients and administered p40HD as above. Whereas injection of p40HD induced strong proliferation of CD8^+^ T cells within low-ischemic allografts from DT-treated wild-type B6 donors, we observed a marked decrease in p40HD-induced CD8^+^ T cell proliferation within allografts from DT-treated B6.CD11c-DTR donors (Figure 3A). Since responsiveness to p40HD requires expression of IL-12Rb1, we compared p40-induced memory CD8^+^ T cell proliferation within heart allografts from wild-type and IL-12Rb1–deficient donors subjected to minimal CIS prior to transplant. These assays showed that the absence of graft IL-12Rb1 totally prevented p40HD-induced memory CD8^+^ T cell proliferation (Figure 3B). Consistent with this, p40HD administration on day 1 after transplant induced IL-15 production in low-ischemic wild-type B6 allografts, whereas DT-mediated depletion of graft CD11c^+^ cells completely abrogated p40HD-induced IL-15 production (Figure 3C). During production IL-15 is often complexed with its specific receptor component, IL-15Rα, and released as a complex into the serum during inflammation (20, 21). Serum levels of IL-15/IL-15Rα complexes were also increased by administration of p40HD to recipients of low-ischemic allografts, and these increases were dependent on CD11c^+^ cells (Figure 3D).

**Figure 4:**
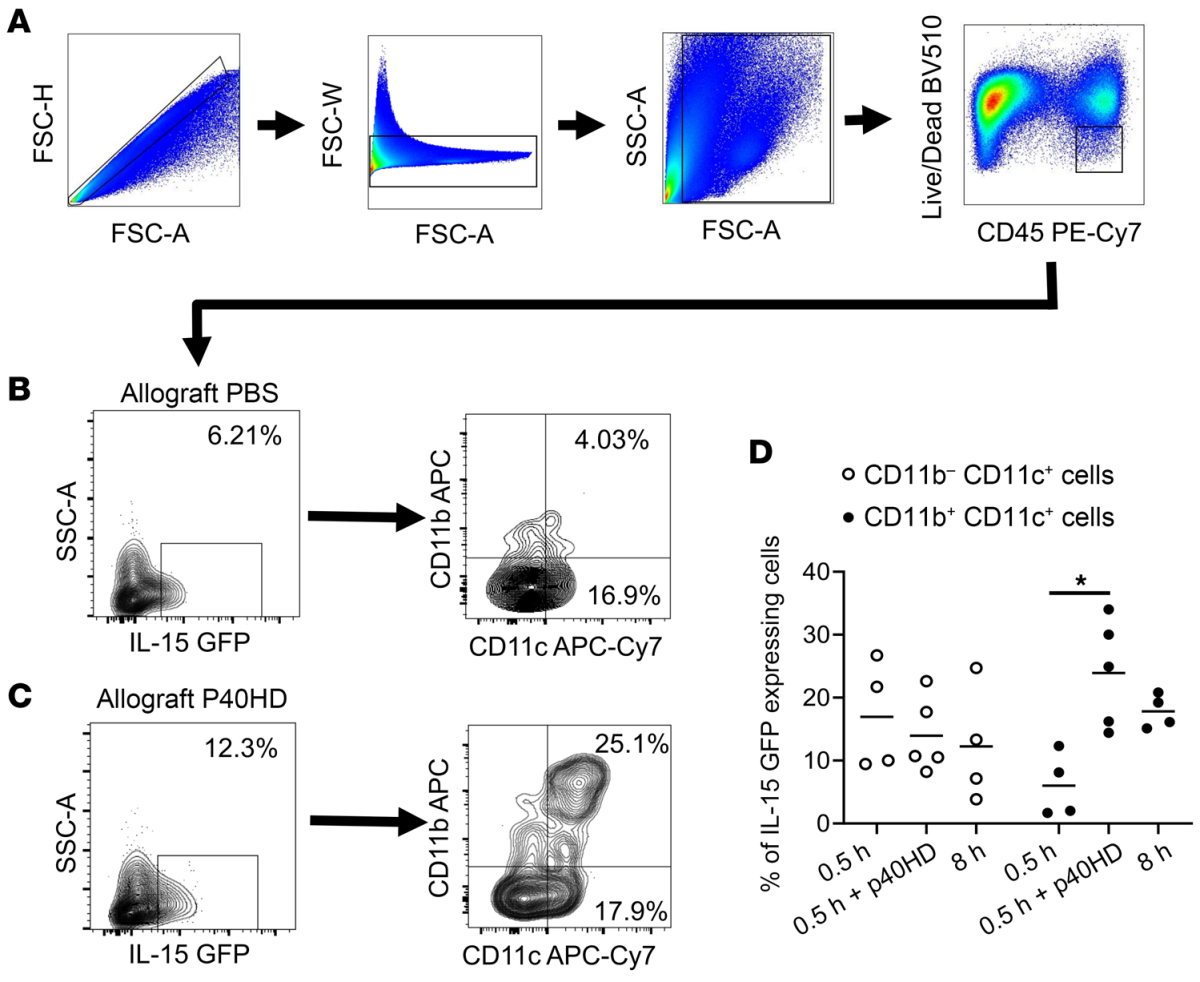
p40HD increases donor-derived DC2 cells’ production of IL-15 within allografts subjected to minimal CIS prior to transplant. (**A**) Groups of BALB/c mice (*n* = 4–5 per group) received cardiac allografts from B6.IL-15 reporter mice subjected to 0.5 or 8 hours of CIS before transplant. Recipients were treated with PBS or 2 μg recombinant p40HDs i.v. on day 0 after transplant. On day 1, allografts were harvested and digested, and cell suspensions were stained with antibody and analyzed by flow cytometry. (**A**–**C**) Gating strategy of IL-15–GFP–expressing myeloid subsets in the allografts of indicated recipients. (**B**–**D**) Representative (**B** and **C**) or pooled (**D**) flow cytometry data of CD11b^+^ and CD11c^+^ cells expressing the IL-15 reporter in allografts of recipients injected with PBS (**B**) or p40HDs (**C**). *P < 0.05 as determined by the Kruskal-Wallis test.

To more precisely identify the IL-15–producing cells within the transplants, we subjected hearts from B6.IL-15 reporter mice to minimal CIS followed by transplant to BALB/c recipients with or without peri-transplant p40HD injection on day 0. The allografts were harvested on day 1 after transplant and digested to prepare single-cell suspensions for flow cytometry, and then viable CD45^+^ cells were analyzed for expression of the IL-15 reporter in the FITC channel (Figure 4A). In p40HD-injected recipients, we observed IL-15^+^ allograft cells within 4 populations of myeloid cells that included 2 DC populations, CD11b^–^CD11c^+^ DC1 cells and CD11b^+^CD11c^+^ DC2 cells (Figure 4, B and C). Since the previous experiments indicated that allograft DCs were required for the IL-15 driving endogenous memory CD8^+^ T cell proliferation in grafts, we focused on these 2 populations. Our analyses showed that in the absence of p40HD, 4% of DC2 cells expressed the IL-15 reporter, but p40HD administration increased IL-15 positivity in DC2 cells approximately 4- to 5-fold. In contrast, approximately 17% of allograft DC1 cells expressed the IL-15 reporter with or without p40HD administration (Figure 4, C and D).

### Blocking CD122 function reverses CTLA-4Ig–resistant rejection of high-risk allografts.

**Figure 5:**
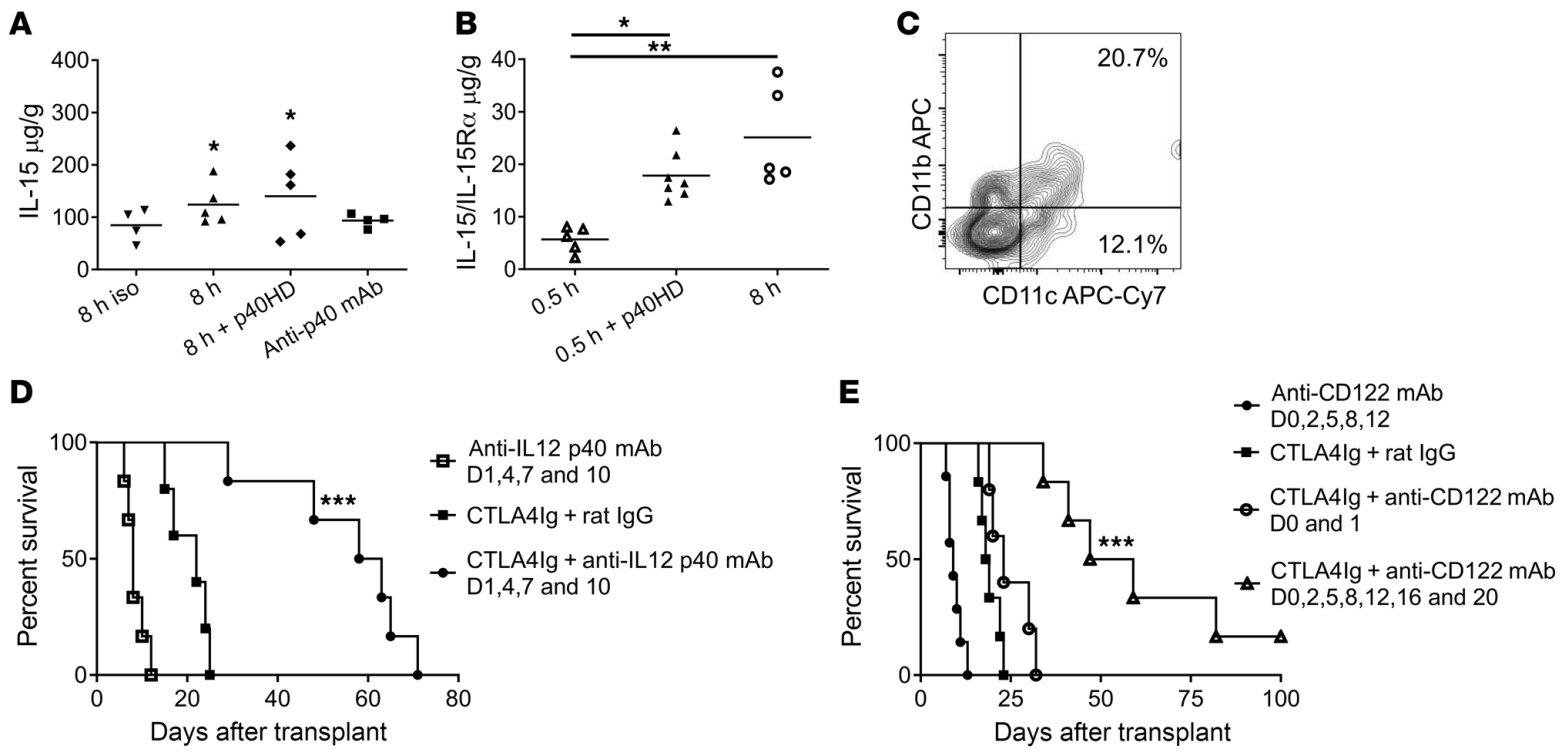
Peri-transplant anti-p40 or CD122 mAb treatment prolongs highly ischemic allograft survival in CTLA-4Ig–conditioned recipients. C57BL/6 (*n* = 4–5 per group) mice received C57BL/6 cardiac isografts or A/J allografts subjected to 0.5 or 8 hours of CIS. The indicated allograft recipients were treated with 2 μg recombinant p40HDs or 200 mg anti-p40 mAb on day 1 after transplant. (**A**) On day 2 after transplant, grafts were harvested and homogenized, and IL-15 was quantified by ELISA. *P < 0.05 vs. isografts, as determined by the Kruskal-Wallis test. (**B**) On day 2 after transplant, grafts were harvested and homogenized, and IL-15/IL-15Rα complexes were quantified by ELISA. *P < 0.05, **P <.01 vs. 0.5-hour-CIS allografts, as determined by the Kruskal-Wallis test. (**C**) BALB/c mice (*n* = 4 per group) received cardiac allografts from B6.IL-15 reporter mice subjected to 8 hours of CIS before transplant. On day 1, allografts were harvested and digested, and cell suspensions were stained with antibody and analyzed by flow cytometry and gated to identify graft-derived IL-15–GFP–expressing myeloid subsets. Representative flow cytometry data of CD11b^+^ and CD11c^+^ cells expressing the IL-15 reporter in high-ischemic allografts are shown, and pooled data are shown in Figure 4D. (**D** and **E**) Survival of A/J allografts subjected to 8 hours of CIS in C57BL/6 recipients treated with or without 250 mg CTLA-4Ig i.p. on days 0 and 1 and given either 200 μg rat IgG, anti-p40 mAb on days 1, 4, 7, and 10 (**D**), or anti-CD122 mAb on days 0 and 1 or days 0, 2, 5, 8, 12, 16, and 20 after transplant (**E**). Graft survival was monitored daily by abdominal palpation, and rejection was confirmed visually by laparotomy. ***P < 0.001 vs. A/J allograft survival in CTLA-4–treated C57BL/6 recipients, as determined by the log-rank (Mantel-Cox) test.

Focusing on high-ischemic allografts, we observed increased production of IL-15 in allografts versus isografts that was blocked by treatment of recipients with anti-p40 mAb (Figure 5A). In contrast to low-ischemic allografts, p40HD did not increase high-ischemic allograft production of IL-15. Production of IL-15/IL-15Rα complexes was also significantly increased in high- versus low-ischemic allografts on day 2 after transplant and was equivalent to the production in low-ischemic allografts from recipients treated with p40HD (Figure 5B). To determine whether the increased IRI-induced inflammation in allografts subjected to prolonged CIS would alter the graft DC populations producing IL-15, we harvested high-ischemic IL-15 reporter allografts from BALB/c recipients and observed the dominant IL-15 expression in the graft DC2 versus DC1 cells similar to that observed in low-ischemic allografts from recipients treated with p40HD (Figure 4D and Figure 5C).

Our previous studies indicated the CTLA-4Ig–resistant activation of endogenous donor-reactive memory CD8^+^ T cells within high-ischemic allografts to mediate acute rejection in CTLA-4Ig–conditioned recipients (16, 17). Whereas anti-p40 mAb did not extend survival of high-ischemic heart allografts in recipients without peri-transplant CTLA-4Ig conditioning (median survival time [MST] 8.0 days), anti-p40 mAb treatment of recipients conditioned with CTLA-4Ig markedly prolonged high-ischemic allograft survival (MST 60.5 days) compared with allograft survival in CTLA-4Ig–conditioned recipients treated with control rat IgG (MST 22 days) (Figure 5D). Consistent with the allograft DC-derived p40HD production required for endogenous donor-reactive memory CD8^+^ T cell activation (Figure 3A), DT-depletion of graft DCs prior to transplant prolonged survival of high-ischemic B6.CD11c-DTR heart allografts in BALB/c recipients conditioned with CTLA-4Ig (MST 22 vs. 39.9 days, *P* < 0.01; Supplemental Figure 4).

Since p40HDs induce IL-15 production in high-ischemic allografts, we also tested whether anti-CD122 mAb treatment extended allograft survival in CTLA-4Ig–conditioned recipients (Figure 5E). Administration of anti-CD122 mAb only on days 0 and 1 was ineffective in extending allograft survival in CTLA-4Ig–conditioned recipients (MST 18.5 vs. 23 days in recipients treated with CTLA-4Ig plus rat IgG). However, a more extensive administration protocol markedly extended survival of high-ischemic allografts versus control IgG-treated recipients conditioned with CTLA-4Ig (MTS, day 53 vs. day 18.5 after transplant), with some allografts surviving beyond 60 days after transplant. Collectively, the results indicate the efficacy of blocking CD122 function to inhibit endogenous memory CD8^+^ T cell proliferation and reversing CTLA-4Ig–resistant rejection to achieve long-term survival of high-ischemic heart allografts.

### Endogenous memory CD8^+^ T cell proliferation within high-ischemic allografts requires IL-15 transpresentation by allograft cells.

**Figure 6:**
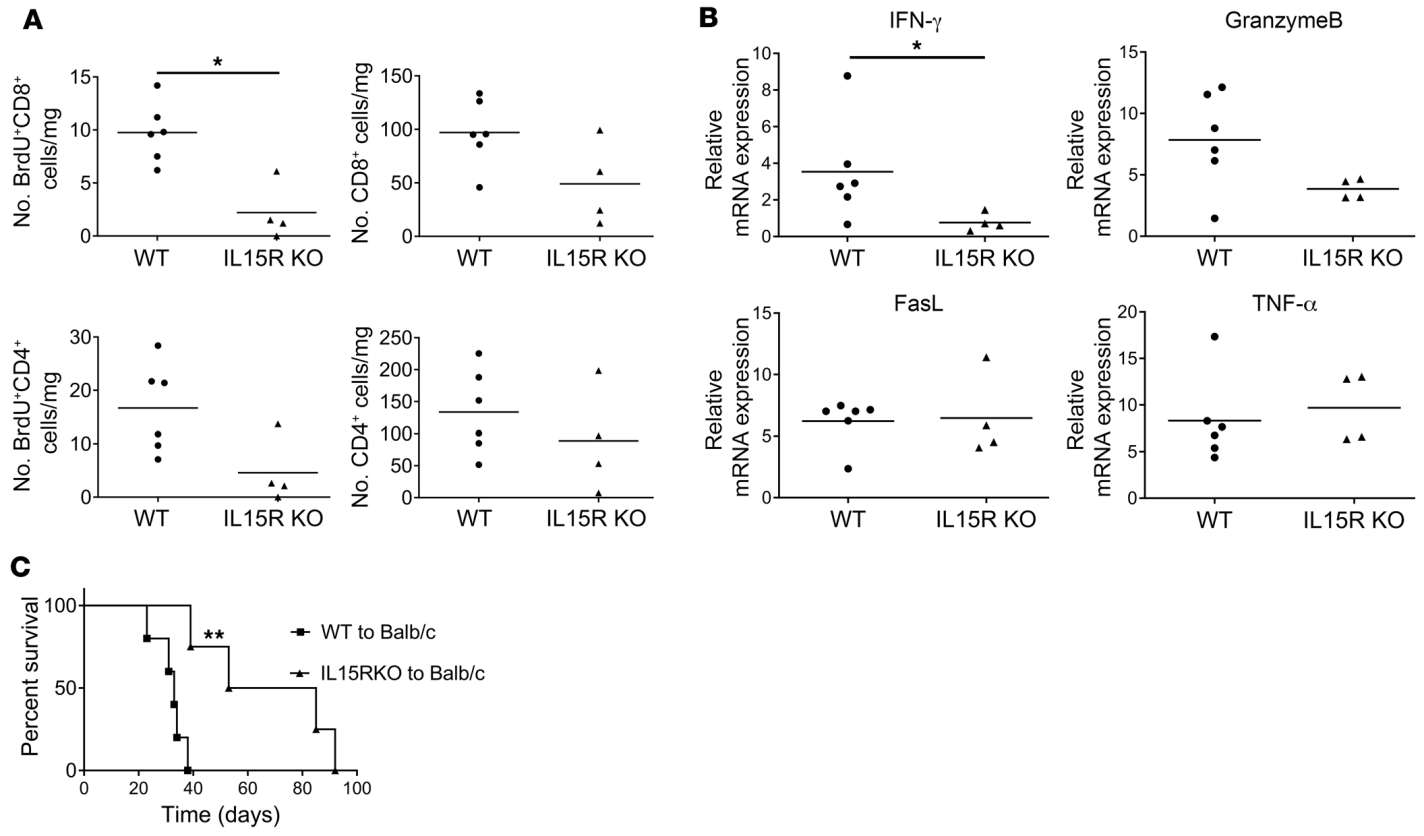
Endogenous memory CD8^+^ T cell proliferation within the allograft subjected to prolonged CIS requires IL-15 transpresentation. (**A**) Groups of BALB/c mice (*n* = 4–6 per group) received cardiac allografts subjected to 8 hours of CIS from either C57BL/6 mice or B6.129X1-IL15ra^tm1Ama^/J mice and were injected with 100 μg BrdU i.p. on days 0 and 1. On day 2 after transplant, allografts were harvested and digested, and aliquots of single-cell suspensions were stained with antibody and analyzed by flow cytometry to quantitate the infiltration and proliferation of memory CD4^+^ and CD8^+^ T cells. *P < 0.05 as determined by the Mann-Whitney nonparametric test. (**B**) Allografts were harvested 48 hours after transplant, and total RNA was isolated from heart graft homogenates from each recipient and analyzed by quantitative reverse transcriptase PCR for expression of the indicated inflammatory mediator gene. Data indicate relative RNA expression of each test mediator versus expression in hearts from non-transplanted C57BL/6 mice. *P < 0.05 vs. RNA expression of C57BL/6 allografts subjected to 8 hours of CIS, as determined by the Mann-Whitney nonparametric test. (**C**) Survival of C57BL/6 or B6.129X1-IL15ra^tm1Ama^/J allografts subjected to 8 hours of CIS in BALB/c recipients conditioned with 250 mg CTLA-4Ig i.p. on days 0 and 1. Graft survival was monitored daily by abdominal palpation, and rejection was confirmed visually by laparotomy. **P < 0.01 vs. survival of C57BL/6 allografts in CTLA-4–treated BALB/c recipients, as determined by the log-rank (Mantel-Cox) test.

IL-15–induced CD8^+^ and NK cell proliferation and homeostasis require IL-15–producing cell transpresentation as an IL-15/IL-15Rα chain complex that is absent in IL-15Rα^–/–^ mice (22–24). Therefore, we tested the requirement for allograft IL-15Rα expression by comparing endogenous memory CD8^+^ T cell proliferation within high-ischemic wild-type C57BL/6 and B6.129X1-IL15Rα^–/–^ allografts subjected to 8 hours of CIS before transplant to BALB/c recipients. There was a marked decrease of proliferating endogenous memory CD8^+^ T cells in the high-ischemic IL-15Rα–deficient versus wild-type allografts (Figure 6A). Consistent with our previous studies indicating that endogenous memory CD4^+^ T cell proliferation within high-ischemic allografts is dependent on memory CD8^+^ T cell activation (17), CD4^+^ T cell proliferation within the highly ischemic IL-15Rα–deficient allografts was also compromised, though the decrease did not achieve significance. The absence of endogenous memory CD8^+^ T cell proliferation within the high-ischemic IL-15Rα–deficient allografts was reflected by the significant decrease in expression of IFN-γ as well as by the decrease in granzyme B expression, which did not achieve statistical significance (Figure 6B), and the abrogation of CTLA-4Ig–resistant rejection in peri-transplant CTLA-4–treated recipients when compared with the CTLA-4Ig–resistant rejection of wild-type allografts (MTS, day 69 vs. 33 after transplant) (Figure 6C). Collectively, these results indicated that allograft cell IL-15 production and transpresentation with IL-15Rα within high-ischemic allografts are required for endogenous donor-reactive memory CD8^+^ T cell proliferation to mediate CTLA-4Ig–resistant rejection of the allografts.

### p40HD-induced memory CD8^+^ T cell proliferation within low-ischemic allografts is not connected to increased effector function.

**Figure 7:**
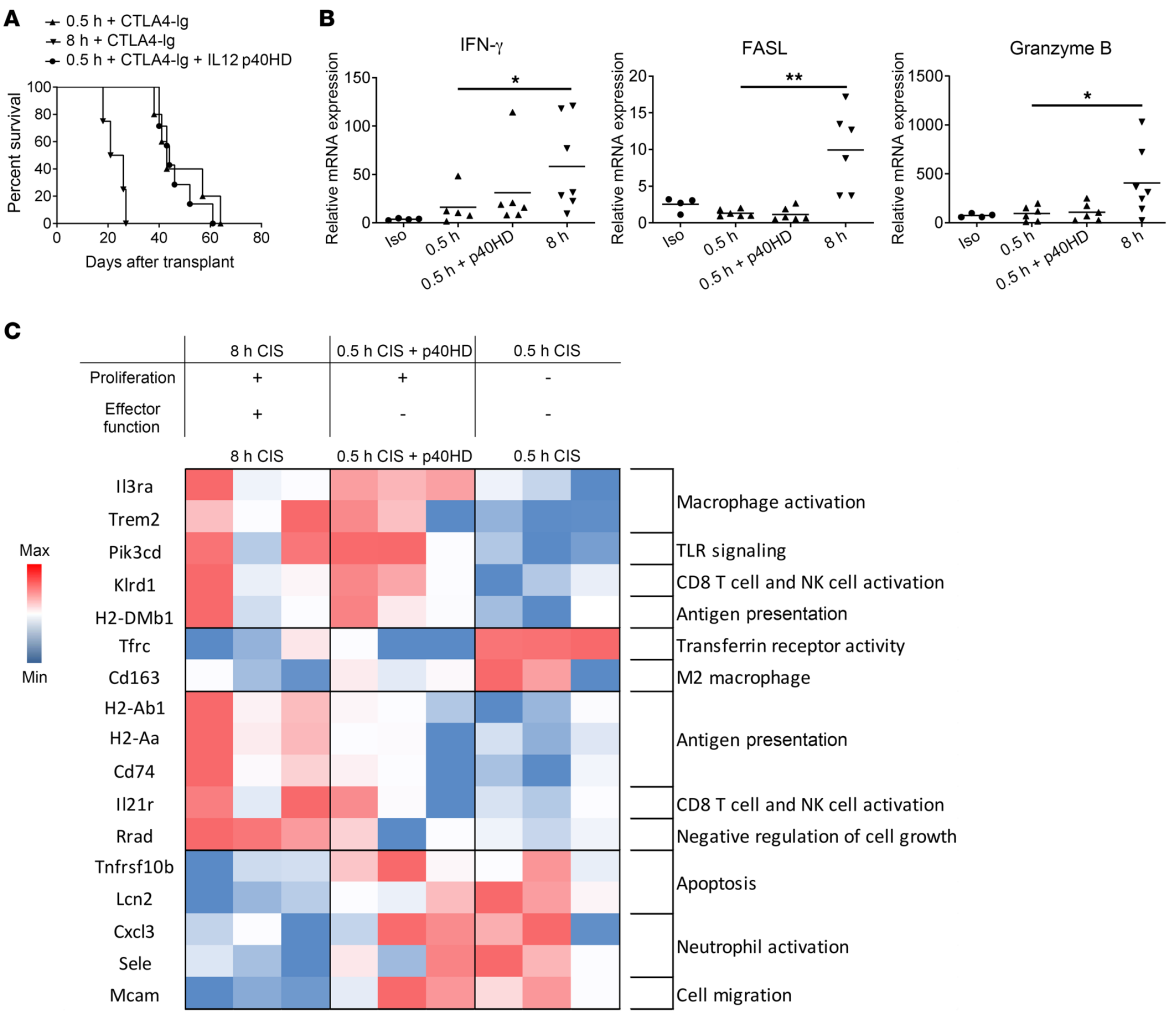
Recombinant p40HDs do not promote CTLA-4Ig–resistant rejection of allografts subjected to minimal CIS. (**A**) Groups of C57BL/6 mice (*n* = 4–7 per group) received A/J allografts subjected to either 0.5 or 8 hours of CIS and were conditioned with 250 mg CTLA-4Ig i.p. on days 0 and 1 and injected with either PBS or 2 μg recombinant p40HDs i.v. on days 1 and 2. Graft survival was monitored daily by abdominal palpation, and rejection was confirmed visually by laparotomy. (**B**) Groups of C57BL/6 mice (*n* = 4–7 per group) received A/J allografts subjected to either 0.5 or 8 hours of CIS and injected with either PBS or 2 μg recombinant p40HDs i.v. on day 1. Grafts were harvested 48 hours after transplant, and total RNA was isolated from heart graft homogenates and analyzed by quantitative reverse transcriptase PCR for expression of the indicated inflammatory mediator genes. Data indicate relative expression versus expression in naive hearts from A/J mice. *P < 0.05, **P < 0.01 vs. the expression of A/J allografts subjected to 0.5 hours of CIS, as determined by the Kruskal-Wallis test. (**C**) Groups of C57BL/6 mice (*n* = 3 per group) received A/J allografts subjected to either 0.5 or 8 hours of CIS and were injected with either PBS or 2 μg recombinant p40HDs i.v. on day 1. Grafts were harvested 48 hours after transplant, total RNA was isolated from heart graft homogenates, and transcript levels were analyzed by NanoString nCounter Gene Expression Assay using the Mouse PanCancer Immune Profiling panel. The heatmap was made in Microsoft Excel using log_2_ normalized counts. Red shaded boxes indicate transcript expression higher than the mean; blue shaded boxes indicate transcript expression lower than the mean.

Since p40HD administration induced IL-15 production and endogenous donor-reactive memory CD8^+^ T cell proliferation within low-ischemic allografts, we tested whether p40HD administration conferred CTLA-4Ig–resistant rejection of low-ischemic allografts. Peri-transplant administration of p40HD on days 1 and 2 after transplant did not abrogate the extended low-ischemic allograft survival in CTLA-4Ig–conditioned recipients (Figure 7A), suggesting that p40-induced proliferation of endogenous memory CD8^+^ T cells was disconnected from their expression of effector function required to mediate allograft injury. In support of this, exogenous p40HD administration to recipients of low-ischemic allografts did not increase allograft expression of IFN-γ, FasL, and granzyme B mRNA (Figure 7B), mediators associated with the endogenous donor-reactive memory CD8^+^ T cell functions observed in the high-ischemic allografts (16, 17).

The ability of exogenous p40HD to induce proliferation of endogenous memory CD8^+^ T cells within low-ischemic allografts without increasing their expression of effector functions observed in high-ischemic allografts predicted that there would be both shared and distinct sets of transcripts expressed in low-ischemic grafts from recipients treated with and without p40HD injection and in high-ischemic allografts where high levels of p40HD are stimulated by the increased IRI. This prediction was tested by interrogation of RNA isolated from allograft tissue on day 2 after transplant using NanoString nCounter analysis. Differentially expressed genes (DEGs) in the high- versus low-ischemic allografts included those involved in TLR signaling, macrophage activation, and CD8^+^ T cell activation (Supplemental Figure 5A). Comparison of DEGs in low-ischemic allografts with and without p40HD stimulation also indicated differences in macrophage activation and TLR signaling, although the upregulated genes associated with TLR signaling were different from those genes involved in TLR signaling that were upregulated in the high-ischemic allografts (Supplemental Figure 5, B and C). DEGs in high-ischemic versus p40HD-stimulated low-ischemic allografts indicated upregulated expression of genes involved in IL-21 receptor signaling and downregulated expression of genes involved in apoptosis. The coordination of DEGs in the 3 sets of allografts at the early time point after transplant indicated shared upregulation of genes involved with macrophage activation, TLR signaling, CD8^+^ T cell activation, and class II MHC antigen presentation in both high-ischemic and p40HD-stimulated low-ischemic allografts versus low-ischemic allografts without p40HD stimulation (Figure 7C). However, DEGs in the high-ischemic allografts indicated differences in decreased expression of genes involved in apoptosis and increased expression of genes involved in memory CD8^+^ T cell activation when compared with expression in low-ischemic allografts with and without p40HD stimulation.

**Figure 8:**
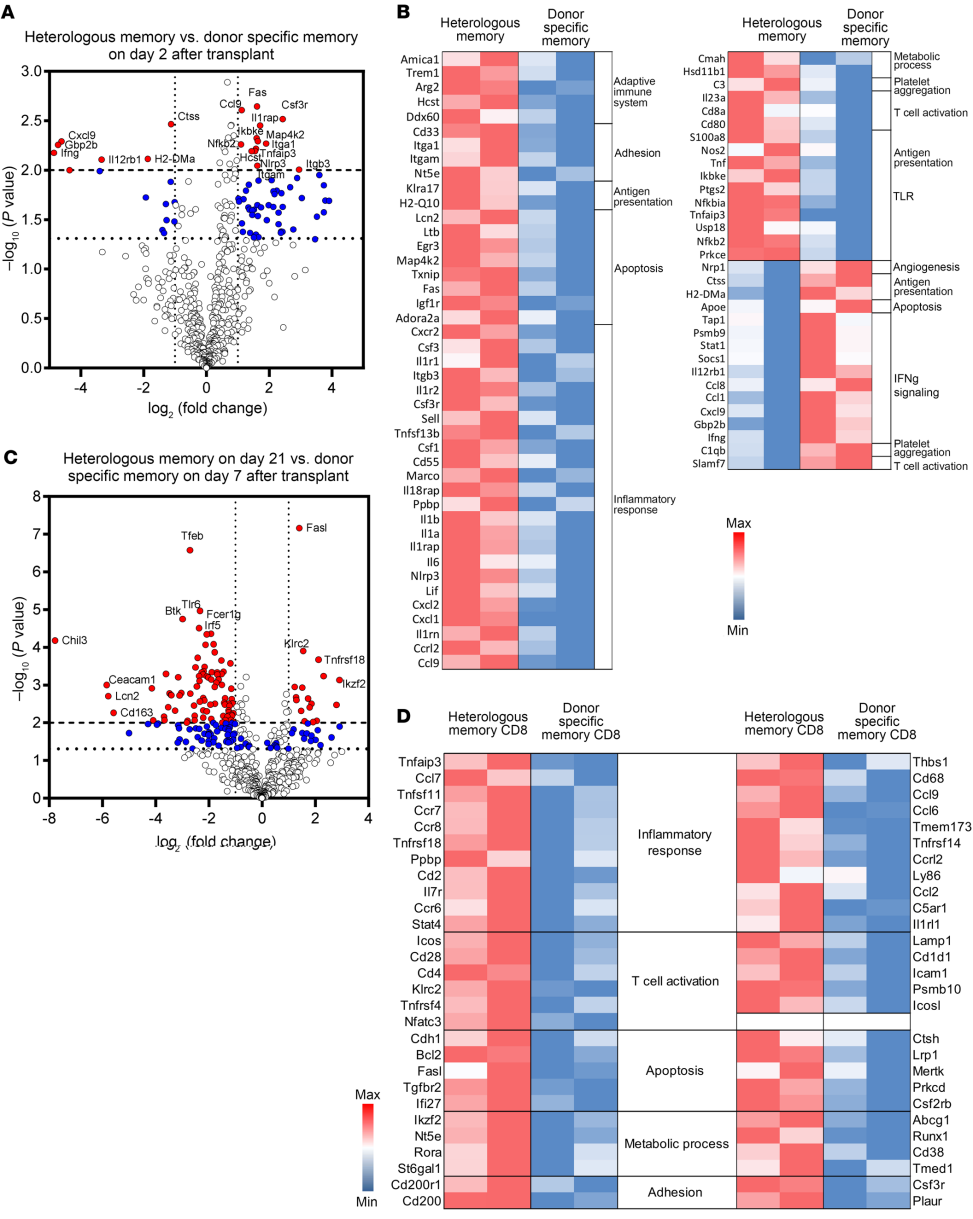
Transcript responses of heterologous and donor-primed memory CD8^+^ T cells infiltrating high-ischemic cardiac allografts. A/J skin allograft–primed CD8^+^ T cells from CD45.2^+^ C57BL/6 recipients were enriched from recipient spleens, and 2 × 10^6^ cell aliquots were transferred to CD45.1^+^ C57BL/6 mice that, 3 days later, received transplanted A/J heart allografts subjected to 8 hours of CIS prior to transplant. CD45.2^+^ C57BL/6 mice also received high-ischemic A/J allografts. All heart allograft recipients were treated with 250 mg CTLA-4Ig i.p. on days 0 and 1. On day 2, allografts were harvested from the B6.CD45.1^+^ and CD45.2^+^ CTLA-4Ig–conditioned recipients (*n* = 2–3) and were digested to obtain single-cell suspensions, cell aliquots were stained with fluorochrome-labeled mAb to identify the CD45.2^+^CD44^+^CD8^+^ heterologous and transferred donor-primed memory CD8^+^ T cells that were purified by flow sorting, and CD8^+^ T cell RNA was analyzed by NanoString using the Mouse PanCancer Immune Profiling panel. (**A** and **C**) Volcano plots indicate DEGs by purified heterologous versus donor-primed memory CD8^+^ T cells infiltrating A/J allografts on day 2 (**A**) or at the time of rejection (day 21 vs. day 7, respectively) (**C**). Filled blue circles indicate DEGs with *P* < 0.05; filled red circles indicate DEGs with *P* < 0.01. The higher dashed horizontal line indicates the cutoff for DEGs with *P* < 0.01, and the lower dotted horizontal line indicates the cutoff for DEGs with *P* < 0.05. (**B** and **D**) Kyoto Encyclopedia of Genes and Genomes analyses of biological pathways were derived from DEGs of the heterologous versus donor-primed memory CD8^+^ T cells infiltrating allografts on day 2 after transplant (**B**) and at the time of graft rejection (day 21 for heterologous vs. day 7 for donor-primed memory CD8^+^ T cells) (**D**). Red shaded boxes indicate transcript expression higher than the mean; blue shaded boxes indicate transcript expression lower than the mean.

The unique pathway stimulating heterologous donor-reactive memory CD8^+^ T cell proliferation and effector differentiation within heart allografts raised questions about potential differences in the activation of donor alloantigen–primed memory CD8^+^ T cells. Donor-primed CD44^hi^ memory CD8^+^ T cells isolated from the spleens of CD45.2^+^ C57BL/6 mice 8 weeks after transplant with A/J skin allografts were transferred to CD45.1^+^ C57BL/6 mice. Three days after the transfer, the CD45.1^+^ recipients received transplanted high-ischemic A/J heart allografts and were treated with peri-transplant CTLA-4Ig. Two days after the heart transplant, the allografts were harvested, and the CD45.2^+^ donor-primed memory CD8^+^ T cells infiltrating the allograft were isolated by flow sorting. In separate groups of high-ischemic heart allograft recipients conditioned with CTLA-4Ig, heterologous CD44^hi^ memory-phenotype CD8^+^ T cells were flow-sorted on day 2 after transplant. RNA was isolated from each of the sorted CD45.2^+^ memory CD8^+^ T cell populations and interrogated using the NanoString nCounter platform. Significant gene expression differences (*P* < 0.01) were observed for 13 upregulated and 6 downregulated genes in the heterologous versus donor-primed memory CD8^+^ T cells (Figure 8A). Upregulated transcripts included those encoding integrin and cell adhesion molecules, whereas downregulated transcripts included those encoding IL-12 receptor β1, IFN-γ, and tissue pathogenesis. These differences were also reflected by biological pathway analyses where the transcripts expressed by the heterologous memory CD8^+^ T cells were enriched for adhesion, apoptosis, TLR-related signals, and inflammatory responses and the transcripts expressed by the antigen-primed memory CD8^+^ T cells were enriched for IFN-γ signaling (Figure 8B).

Finally, the heterologous and transferred donor-primed CD45.2^+^ memory CD8^+^ T cells were isolated from high-ischemic allografts in CTLA-4Ig–conditioned recipients at the time of graft rejection, day 21 for the heterologous memory CD8^+^ T cells and day 7 for the donor-primed memory CD8^+^ T cells, and the transcript responses were compared. As with the day 2 responses, marked differences were observed when transcripts from the isolated graft-infiltrating heterologous memory CD8^+^ T cells at rejection were compared with those from the donor-primed memory CD8^+^ T cells, with 17 DEGs significantly upregulated by the heterologous memory CD8^+^ T cells (*P* < 0.01) and 85 DEGs significantly downregulated (*P* < 0.01) (Figure 8C). These differences were reflected by differences in DEGs expressed in specific biological pathways, including those involved in inflammatory responses, T cell activation, apoptosis, and cell adhesion (Figure 8D; and complete DEG data are provided in Supplemental Figure 6).

## Discussion

The low-affinity/degenerate nature of T cell receptor binding to peptide/self MHC complexes coupled with the lower threshold of epitope and costimulation expression required to elicit memory T cell responses often results in memory T cell functional cross-reactivity to peptide/MHC complexes that are distinct from those originally generating the memory T cells (9–11, 25, 26). Heterologous memory T cell immunity is often an important component of responses to MHC-disparate allografts in unsensitized recipients and a risk factor for poorer graft outcomes (27–32). Seminal translational studies of unsensitized kidney transplant patients demonstrated that high pre-transplant frequencies of donor-reactive memory T cells producing IFN-γ are associated with marked increases in acute rejection episodes and decreased graft function during the first year after transplant despite the use of standard-of-care calcineurin-based immunosuppression (27, 30). This clinical experience suggests the likelihood that these memory T cells first infiltrate and then proliferate within the graft to mediate injury. Mechanisms driving proliferation of heterologous antigen-reactive memory CD8^+^ T cells within inflammatory microenvironments are poorly understood. We have reported increases in proliferation of endogenous donor-reactive memory CD8^+^ T cells and their expression of effector function within allografts subjected to prolonged versus minimal CIS immediately after transplant to unsensitized recipients (16, 17). In contrast, preclinical models investigating memory T cells directly generated to donor alloantigens through recipient sensitization document activation in low-ischemic allografts to mediate costimulatory blockade–resistant graft rejection (33–36). These observations suggest different requirements for activation within the allograft to mediate rejection by heterologous donor-reactive memory T cells in unsensitized recipients versus donor alloantigen–primed memory T cells in sensitized recipients. This is supported in the current report by the different transcript responses of heterologous versus donor-primed memory CD8^+^ T cells infiltrating high-ischemic heart allografts and the differences in time to mediate allograft rejection.

Effector memory T cell responses to infection and during autoimmune disease occur in highly inflammatory tissue microenvironments that make the linkage between activation signals to proliferate and signals to express effector function difficult to distinguish. The unique transplant model in which the intensity of early ischemic inflammation in heart allografts is conferred by the length of CIS prior to transplant allowed us to determine the separation of intragraft signals inducing proliferation and expression of effector functions to donor-reactive memory CD8^+^ T cells within the low- and high-ischemic inflammatory graft microenvironment. The ability of exogenously administered p40HD to provoke the memory CD8^+^ T cell proliferation in low-ischemic allografts not only allowed distinction between memory CD8^+^ T cell proliferation and effector function expression but also revealed the mechanism of p40HD-induced proliferation of the memory CD8^+^ T cells.

A key finding of our studies is that stimulation of proliferation of endogenous donor-reactive memory CD8^+^ T cells and their activation to express the effector functions mediating acute graft injury can be separated into 2 distinct events. We used both low-ischemic allografts from recipients injected with exogenous p40HD and high-ischemic allografts to investigate mechanisms underlying heterologous donor-reactive memory CD8^+^ T cell proliferation within the allograft. While increased proliferation and expression of effector function are absent in low-ischemic allografts, p40HD stimulation provoked marked endogenous memory CD8^+^ T cell proliferation via increased IL-15 production without increasing expression of effector functions and the ability to mediate CTLA-4Ig–resistant allograft rejection. In vitro studies have indicated a similar separation where stimulation through 4-1BB promotes proliferation of naive antigen-specific CD4^+^ or CD8^+^ T cells without their differentiation into cytolytic effector cells and CD27 signaling promotes IL-2–independent proliferation of antigen-stimulated CD8^+^ T cells without differentiation into IFN-γ–producing and cytolytic T cells (37, 38). The increased endogenous donor-reactive memory CD8^+^ T cell proliferation within higher ischemic allografts requires graft production of p40HD, but not IL-12 p35/p40 or IL-23 p19/p40 heterodimers. Several bacterial infections induce p40HDs that bind IL-12Rb1 dimers on myeloid cells to stimulate chemokine production and enhance phagocytic function (39–42), but p40HDs have not been reported to impact naive or memory CD8^+^ T cell activation, including stimulation of proliferation.

The uncoupling of proliferation and expression of effector function by endogenous memory CD8^+^ T cells in low-ischemic allografts from p40HD-treated recipients indicates the requirement for the increased inflammation in the higher ischemic allografts to provoke heterologous donor-reactive memory CD8^+^ T cell effector function. This is supported by recent results from our group indicating that the heterologous donor-reactive memory CD8^+^ T cells infiltrating heart allografts subjected to prolonged CIS produce TNF-α mediating RIPK1-dependent graft cell death that is required for CTLA-4Ig–resistant rejection (43). The transcript landscape in allografts subjected to prolonged CIS versus minimal CIS with or without p40HD stimulation reflects the gene expression differences in allografts with minimal donor-reactive memory CD8^+^ T cell activation, allografts with memory CD8^+^ T cell proliferation without expression of effector functions to mediate acute injury, and allografts with both memory CD8^+^ T cell proliferation and strong expression of effector function to mediate acute graft injury. One component of this increased ischemic environment is likely to involve the distinct Toll-like receptor (TLR) signaling pathways observed in transcripts from the high-ischemic allografts. Similarly, high expression of IL-21R gene was observed in the high- but not the low-ischemic allografts with or without p40HD stimulation. IL-21 is of particular interest as it is primarily produced by CD4^+^ T cells and within inflammatory tissue sites promotes the effector functions of CD8^+^ T cells and NK cells (44, 45).

A second key finding of this study is that the p40HD-mediated proliferation of endogenous memory T cells within the higher- and lower-ischemic cardiac allografts occurs indirectly through stimulation of IL-15 production. During inflammatory responses many cytokines and other stimuli directly induce IL-15 production, including type I IFN, GM-CSF, oxidative stress, and TLR signaling (46–48). Although IL-15 production is observed during IRI of many organs (49, 50), mechanisms inducing this production and, specifically, a role for p40HD have not been previously identified. The p40HD-induced proliferation of graft-infiltrating memory CD8^+^ T cells occurred in the low-risk allografts, but not isografts, in recipients injected with p40HD, further indicating the requirement for alloantigen recognition (16). IL-15 is crucial for memory CD8^+^ T cell and NK cell maintenance and proliferation, primarily via transpresentation in a complex with IL-15Rα by the IL-15–producing cell (22–24). Depletion of allograft CD11c^+^ cells from low-ischemic allografts abrogated both p40HD-induced IL-15 production and proliferation of the graft-infiltrating memory CD8^+^ T cells. Recent studies from the Pober laboratory have documented alloantibody-stimulated human endothelial cell production and transpresentation of IL-15/IL-15Rα complexes in vitro and in human aortic allografts placed into mice (51). However, there is little, if any, donor-specific antibody produced during the rapid heterologous memory T cell response to the heterotopically transplanted heart allografts, which may account for graft DCs, rather than endothelial cells, as the primary source of p40HD-induced IL-15.

The p40HD-induced IL-15 that stimulates endogenous memory CD8^+^ T cell proliferation appears to be produced by a specific population of allograft-resident DCs. Whereas low frequencies of allograft CD11c^+^CD11b^–^ DCs produced IL-15 in the low-ischemic allografts with or without exogenous p40HD, exogenous p40HD increased allograft CD11c^+^CD11b^+^ DCs producing IL-15 more than 5-fold. Furthermore, CD11c^+^CD11b^+^ DCs were also at much higher frequencies than CD11c^+^CD11b^–^ DCs in allografts subjected to prolonged CIS prior to transplant, suggesting that IRI regulates the populations and functions of heart allograft–resident DCs. A key function of CD11c^+^CD11b^–^ DCs is acquisition of antigen and cross-presentation to antigen-reactive CD8^+^ T cells (52–54), but such cross-presentation is likely not to be necessary in the heart allografts with most graft cells expressing donor class I MHC alloantigens that are the target ligand of the infiltrating heterologous memory CD8^+^ T cells (15). The increase in IL-15–producing CD11c^+^CD11b^+^ cells implicates these cells as driving p40-induced endogenous memory CD8^+^ T cell proliferation within the allografts, but does not negate a potential role of CD11c^+^CD11b^–^ DCs in this response, as recent studies have shown that CD11c^+^CD11b^–^ DCs promote CD11c^+^CD11b^+^ DC function through production of indoleamine 2,3-dioxygenase-1 and other mediators (55).

Overall, these results identify key targets to inhibit heterologous donor-reactive memory CD8^+^ T cell activation within the allograft microenvironment and improve survival of allografts subjected to longer CIS times prior to transplant, such as deceased-donor grafts. Short courses of anti-p40 and anti-CD122 mAb resulted in marked prolongation of the allografts when used in recipients conditioned with a short peri-transplant course of CTLA-4Ig. Mathews and colleagues have also reported that the combination therapy of anti-CD122 and CTLA-4Ig significantly prolonged kidney allograft survival in nonhuman primates and inhibited both proliferation and the expression of effector memory CD8^+^ T cell function (35). The results of the current study also suggest that components of the IRI response might be effective new targets in attenuating the inflammatory environment driving donor-reactive memory CD8^+^ T cell expression of effector function to mediate acute graft injury. Increased IRI is an important risk factor undermining early graft function and long-term outcomes of clinical organ transplants (56–58), and our results implicate a direct connection between heterologous memory T cell–mediated alloimmunity and IRI.

## Methods

The general experimental design and tools are depicted in Supplemental Figure 7.

### Mice.

C57BL/6 (H-2^b^), B6.diphtheria toxin receptor–CD11c (DTR-CD11c) transgenic mice, B6.129X1-IL15ra^tm1Ama^, B6.129S1-IL12rb1^tm1Jm^, CD45.1^+^ B6.SJL-PtprcaPepcb/BoyJ, A/J (H-2^a^), and BALB/c (H-2^d^) mice were purchased from The Jackson Laboratory and maintained under standard specific pathogen–free conditions. IL-15 translational GFP reporter mice were produced by Tomas Sosinowski and provided by Ross Kedl, University of Colorado (Denver, Colorado, USA) (24), and were bred in-house at the Lerner Research Institute.

### Heterotopic cardiac transplantation.

Intra-abdominal heterotopic cardiac transplantation was performed as adapted from the method established by Corry and colleagues (15, 59). Allogeneic and syngeneic donor hearts were harvested and stored for either 0.5 or 8 hours in cold Ringer’s solution (catalog R5310-01, B. Braun Medical Inc.) before the donor aorta and pulmonary artery were anastomosed to the recipient abdominal aorta and inferior vena cava in the peritoneal cavity. Graft survival was monitored daily by recipient abdominal palpation, and rejection was visually confirmed by laparotomy.

### In vivo antibody treatment.

CD4^+^ T cell depletion in graft recipients was performed using a 1:1 cocktail of anti-CD4 mAb (catalog BE0119 and BE0003-1, clone YTS191 and GK1.5, Bio X Cell) and 0.2 mg given i.p. on days –3, –2, and –1 before transplant. CD4^+^ T cell depletion was at least 98% in peripheral blood and spleen in treated sentinel animals. CTLA-4Ig (catalog BE0099, clone CTLA-4, Bio X Cell) was given at a daily dose of 0.25 mg i.p. on days 0 and +1 after transplant. Anti-p40 mAb (catalog 505305, clone C17.8, BioLegend) was given at 0.2 mg i.p. daily on days 0 and +1. Anti-CD122 (catalog BE0272, clone 5H4, Bio X Cell) and anti-CD25 (catalog BE0012, clone PC-61.5.3, Bio X Cell) mAbs were injected at 0.1 mg i.p. daily on days 0 and +1. In all cases, equal amounts of normal rat IgG (catalog I4131, MilliporeSigma) were administered to control allograft recipient groups. Recombinant p40 homodimers (catalog 573104, BioLegend), recombinant p35/p40 heterodimers (catalog 577002, BioLegend), or recombinant p19/p40 heterodimers (catalog 589004, BioLegend) were given at 2 μg i.v. on day +1 after transplant. To deplete CD11c^+^ cells, diphtheria toxin (DT; catalog D0564, MilliporeSigma) was injected i.p. (40 ng/g body weight) into B6.DTR-CD11c transgenic and control wild-type mice on days 2 and 1 before transplantation.

### BrdU labeling and flow cytometry analysis of graft-infiltrating CD4^+^ and CD8^+^ T cells.

Mice were injected i.p with 100 μg BrdU (catalog 559619, BD Biosciences) on days 0 and +1. Flow cytometric detection of graft-infiltrating cells was performed using a modification of the method of Afanasyeva and colleagues (15–17, 60). On day 2, harvested grafts were weighed, minced, and incubated for 1 hour at 37°C in RPMI medium with type II collagenase (catalog 1005021, MP Biomedicals) and DNase I (catalog 10104159001, MilliporeSigma). The digested graft was pressed with the plunger of a 3 cc syringe, and after 40 μm filtration and centrifugation, the collected cells were counted and aliquots were incubated with CD16/32 mAb (catalog 553142, BD Biosciences) and stained with the following antibodies: PE–anti-CD4 (catalog 553049, clone RM4-5, BD Biosciences), APC–anti-CD8 (catalog 553035, clone 53-6.7, BD Biosciences), PE-Cy7–anti-CD45 (catalog 20-0451-81, clone 30-F11, Thermo Fisher Scientific), PE–anti-CD122 (catalog 105905, clone 5H4, BioLegend), PE–anti–IL-15R (catalog 12-7149-82, clone DNT15Ra, Thermo Fisher Scientific), APC–anti-CD25 (catalog 17-0251-82, clone PC61.5, Thermo Fisher Scientific), and APC–anti-CD127 (catalog 135011, clone A7R34, BioLegend). Intracellular staining to detect BrdU incorporation was performed using the FITC BrdU Kit according to the manufacturer’s protocol (catalog 559619, BD Biosciences). Analyses were performed on an LSR II or LSR Fortessa X20 flow cytometer (BD Biosciences), and data analyses were performed by FlowJo software version 10 (Tree Star Inc.). Total numbers of each leukocyte population were determined by: (the total number of CD45^+^ leukocytes counted) × (% of the target leukocyte population in the CD45^+^ cells)/100. These data are reported as number of each leukocyte population per milligram graft tissue. The gating strategy for identifying proliferating CD8^+^ cells in the graft on day 2 after transplant is shown in Figure 1A. After exclusion of doublets (FSC-H vs. FSC-A) and debris (using SSC-A vs. FSC-A), the cells were first gated on the CD45^+^ leukocyte population, and CD8^+^ T cells were analyzed for uptake of BrdU. For analysis of the IL-15 reporter expression by cells in the graft, single-cell suspensions from harvested grafts were prepared and stained with PE-Cy7–anti-CD45 (catalog 20-0451-81, clone 30-F11, Thermo Fisher Scientific), APC–anti-CD11b (catalog 17-0112-82, clone M1/70, Thermo Fisher Scientific), and APC-Cy7–anti-CD11c (catalog 117352, clone N418, BioLegend) antibodies, followed by BV510–dead cell stains using LIVE/DEAD Fixable Dead Cell Stain kits (catalog MP34955, Thermo Fisher Scientific).

### RNA extraction and analysis.

Total RNA was extracted from harvested grafts using RNeasy Fibrous Tissue Kits (catalog 74704, QIAGEN) and reverse-transcribed to cDNA using High-Capacity cDNA Reverse Transcription Kits (catalog 4368814, Applied Biosystems). CD8^+^ T cells infiltrating grafts on day 2 after transplant were enriched using a negative selection mouse T cell isolation kit (catalog 19853, STEMCELL Technologies), and RNA was isolated using the RNeasy Micro Kit (catalog 74004, QIAGEN) and cDNA generated followed by pre-amplification using 2× Pre-amplification Master Mix (catalog 4488593, Applied Biosystems). Gene expression was measured by quantitative PCR using a 7500 Fast Real-Time Thermocycler (Applied Biosystems) with TaqMan Fast Universal Master Mix (catalog 4352042, Applied Biosystems) and TaqMan primer sets (CD25, Mm01340213_m1; CD122, Mm00434268_m1; IL-7R, Mm00434295_m1; IL-15RA, Mm04336046_m1; IFN-γ, Mm01168134_m1; FasL, Mm00438864_m1; granzyme B, Mm00442837_m1; and MRPL32, Mm00777741_sH). Data were normalized to expression of Mrpl32 and RNA isolated from either naive A/J heart or CD8^+^ T cells purified from the spleen of naive A/J mice as the calibrator.

Total allograft RNA was also interrogated by the NanoString nCounter platform by hybridizing to the mouse PanCancer Immune Panel for processing on the nCounter GEN2 Analysis System using the high-sensitivity protocol and high-resolution data capture. The fluorescent barcoded probes of each target RNA molecule were counted by nCounter Digital Analyzer. Raw counts were normalized and analyzed using nSolver version 4.0 software. To identify genes that were significantly differentially expressed, cutoffs of *P* ≤ 0.05 were selected and defined at a threshold of a 2-fold change.

### IL-15 and IL-2 ELISA.

Harvested grafts were immediately snap-frozen and homogenized with Pierce RIPA buffer (catalog 89900, Thermo Fisher Scientific) containing protease inhibitor cocktail (catalog 11836170001, MilliporeSigma). The tissue suspension was centrifuged at 16,100*g* for 20 minutes at 4°C, and the supernatants were collected. The protein concentration of lysates was measured using Coomassie Plus Protein Assay reagent (catalog 23238, Thermo Fisher Scientific). IL-15 and IL-2 levels were quantified by enzyme immunoassay using the Mouse IL-15 or IL-2 DuoSet ELISA kit (catalog DY447-05 and DY402-05, R&D Systems), and IL-15/IL-15R levels were measured using Mouse IL-15/IL-15R ELISA kit (catalog BMS6023, Thermo Fisher Scientific). All values were standardized by the total protein concentration of each sample.

### Donor antigen–primed memory CD8^+^ T cell induction and transfer to heart allograft recipients.

To generate donor antigen–primed memory CD8^+^ T cells, CD45.2^+^ C57BL/6 mice received A/J skin allografts, and 8 weeks later, recipient spleen suspensions were prepared, total CD8^+^ T cells were enriched by a negative selection mouse T cell isolation kit (STEMCELL Technologies), and 2 × 10^6^ aliquots of the purified CD8^+^ T cells were transferred i.v. to congenic CD45.1^+^ C57BL/6 mice. After 72 hours, A/J heart allografts subjected to prolonged CIS were transplanted into the CD45.1^+^ C57BL/6 mice, and 250 μg CTLA-4Ig was injected i.p. on days 0 and +1. The A/J allografts were harvested on day 2 after transplant and digested to prepare single-cell suspensions that were stained with FITC–anti-CD45.2 (catalog 553772, clone 104, BD Biosciences), Pacific blue–anti-CD8 (catalog 558106, clone 53-6.7, BD Biosciences), and APC-CD44 mAb (catalog 17-0441-82, clone IM7, Thermo Fisher Scientific). The graft-infiltrating CD45.2^+^CD44^+^CD8^+^ T cells were purified by cell sorting using the BD Aria III sorter (BD Biosciences) and were resuspended in a lysis solution of 1 part RLT buffer from the QIAGEN RNeasy Fibrous Kit to 2 parts nuclease-free water, and 4.5 μL of the lysate was added directly to the mouse PanCancer Immune Panel code set and then processed on the nCounter GEN2 Analysis System using the high-sensitivity protocol and high-resolution data capture.

### Statistics.

All data were analyzed using GraphPad Prism Pro version 10 (GraphPad Software Inc.). Numbers in experimental groups were chosen using power analyses from previously performed experiments. Comparisons between experimental and control or naive groups were determined by the Mann-Whitney nonparametric test or nonparametric equivalent of a 1-way ANOVA, the Kruskal-Wallis test. Heart allograft survival was plotted using Kaplan-Meier cumulative survival curves, and differences in survival between groups were evaluated by the log-rank (Mantel-Cox) test. *P* less than 0.05 was considered to be statistically significant. All values for experimental groups are expressed as mean ± SEM.

### Study approval.

All animal studies were conducted on male mice between 8 and 12 weeks of age and were approved by the Institutional Animal Care and Use Committee of the Cleveland Clinic. Use of diphtheria toxin was approved by the Institutional Biosafety Committee of the Cleveland Clinic.

### Data availability.

The gene expression data presented in Figures 7 and 8 can be found in the NCBI’s Gene Expression Omnibus database (GEO GSE229880, https://www.ncbi.nlm.nih.gov/geo/query/acc.cgi?acc=GSE229880; and GSE233620, https://www.ncbi.nlm.nih.gov/geo/query/acc.cgi?acc=GSE233620).

## Author contributions

HT and KSK designed and performed the experiments and prepared figures. HT, WMB, PSH, AV, and RLF analyzed data and wrote the manuscript.

## Supplementary Material

Supplemental data

Supporting data values

## Version 1 01/25/2024

In-Press Preview

## Version 2 03/15/2024

Electronic publication
