# Supplementary material for: p40 homodimers bridge ischemic tissue inflammation and heterologous alloimmunity in mice via IL-15 transpresentation
Source: J Clin Invest. 2024 Jan 25;134(6):e172760. doi: 10.1172/JCI172760 (PMC10940089; doi:10.1172/JCI172760)
Supplement: Supplemental data [file jci-134-172760-s250.pdf]

SUPPLEMENTAL FIGURES:

Supplemental Figure 1: Overview of previous results testing the impact of ischemia-reperfusion injury on the activation of heterologous donor-reactive memory CD8 T cells.

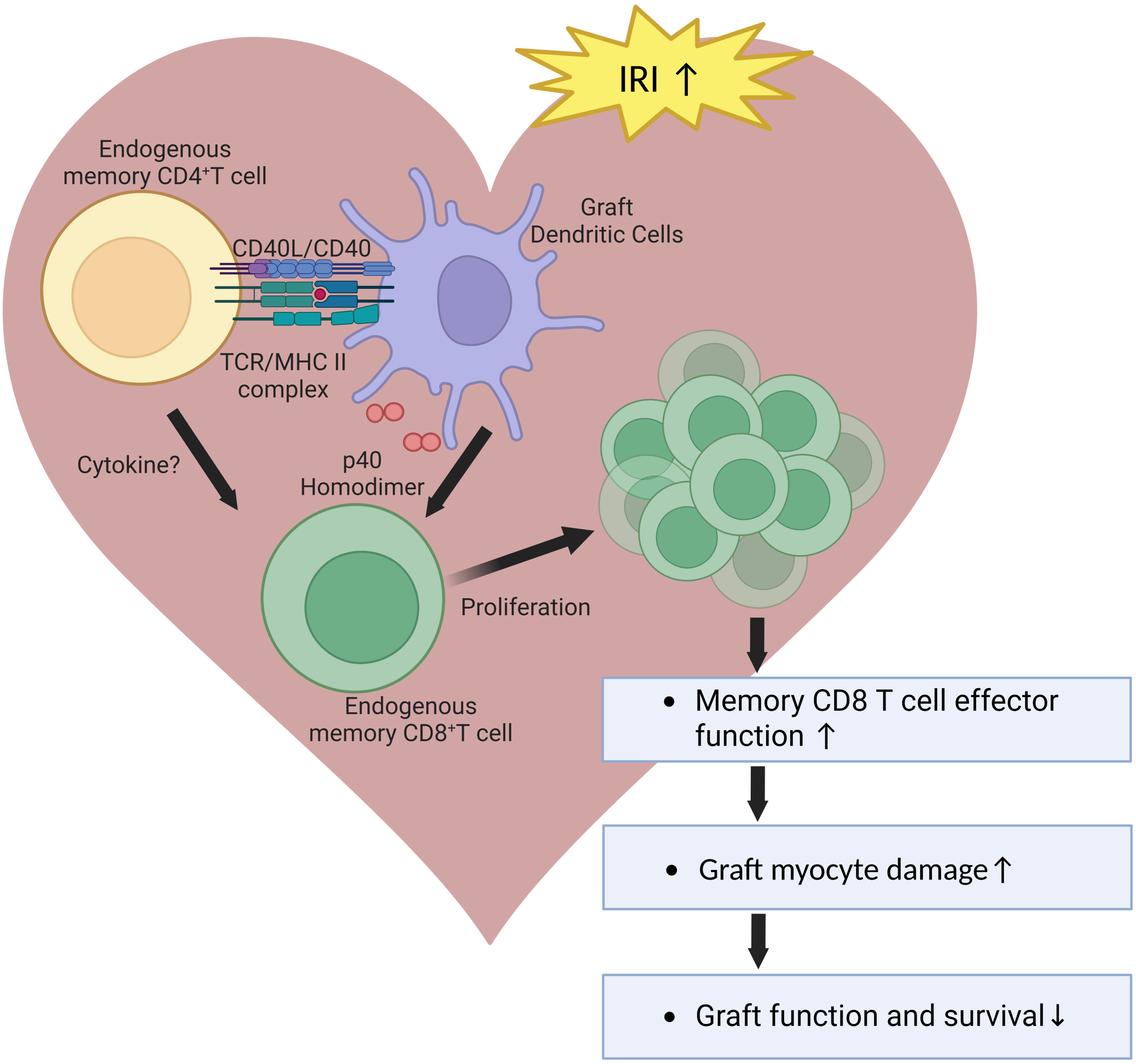

Previous studies demonstrated that increasing the intensity of ischemia-reperfusion injury in heart allografts by subjecting the grafts to prolonged cold ischemic storage before transplant increased donor-reactive memory CD8 T cell proliferation within the allograft on day 2 post-transplant and conferred the ability of these memory CD8 T cells to mediate CTLA-4Ig resistant graft rejection (Reference 16). The activation of these donor-reactive memory CD8 T cells on day 2 post-transplant required help provided by graft

infiltrating recipient CD4 T cell engagement of allogenic class II MHC and CD40 on donor graft-derived dendritic cells that generated p40 dimers required for the memory CD8 T cell proliferation within the allograft.

Supplemental Figure 2. IL12Rβ1 expression on BrdU⁺ CD8⁺ cells infiltrating allografts subjected to 0.5 or 8hrs cold ischemic storage.

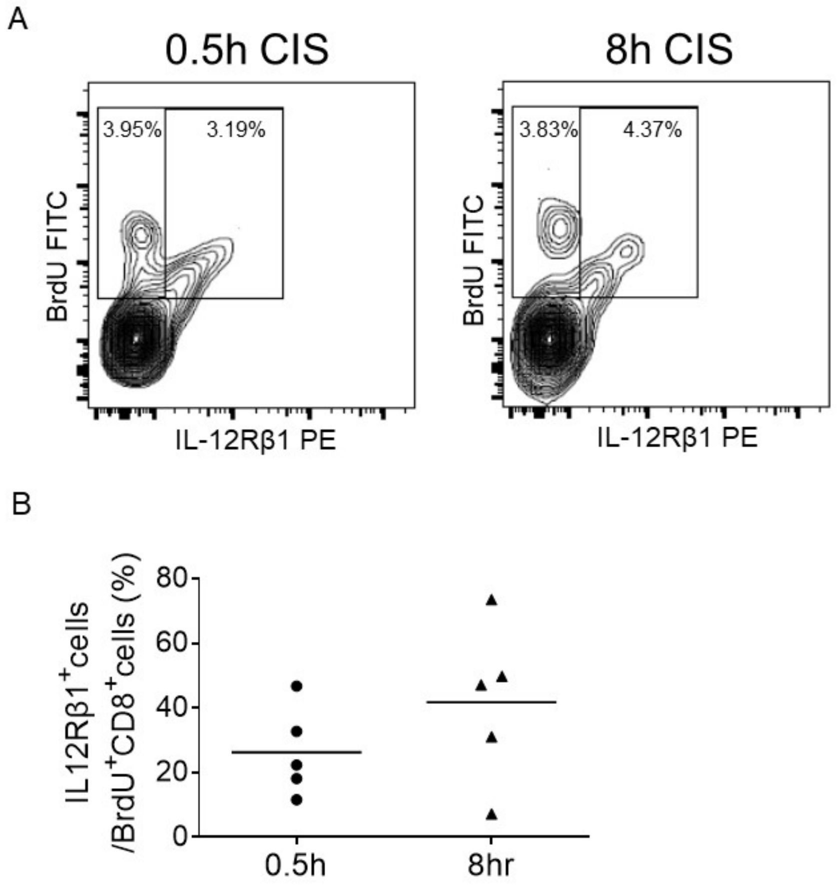

C57BL/6 mice were transplanted with A/J cardiac allografts subjected to either 0.5 or 8 hours of cold ischemic storage (CIS) prior to transplant. All recipients were injected with 100 μg BrdU i.p. on days 0 and 1 after transplant. On day 2 post-transplant, allografts were harvested, digested to prepare single cell suspensions, and aliquots were stained with antibody and analyzed by flow cytometry using the gating strategy shown to assess the proliferation of infiltrating $CD8^+$ T cells in allografts subjected each of the CIS conditions. (A) Representative flow cytometry data of IL12Rβ1 expression on $BrdU^+$ and $BrdU^-$ $CD8^+$ cells in the allograft. (B) Percentages of IL12Rβ1 expressing $BrdU^+$ $CD8^+$ cells in groups of 5 A/J allografts subjected to 0.5 or 8 hours of CIS.

Supplemental Figure 3: CD122 is upregulated in proliferating CD8 T cells within the allografts subjected to prolonged CIS.

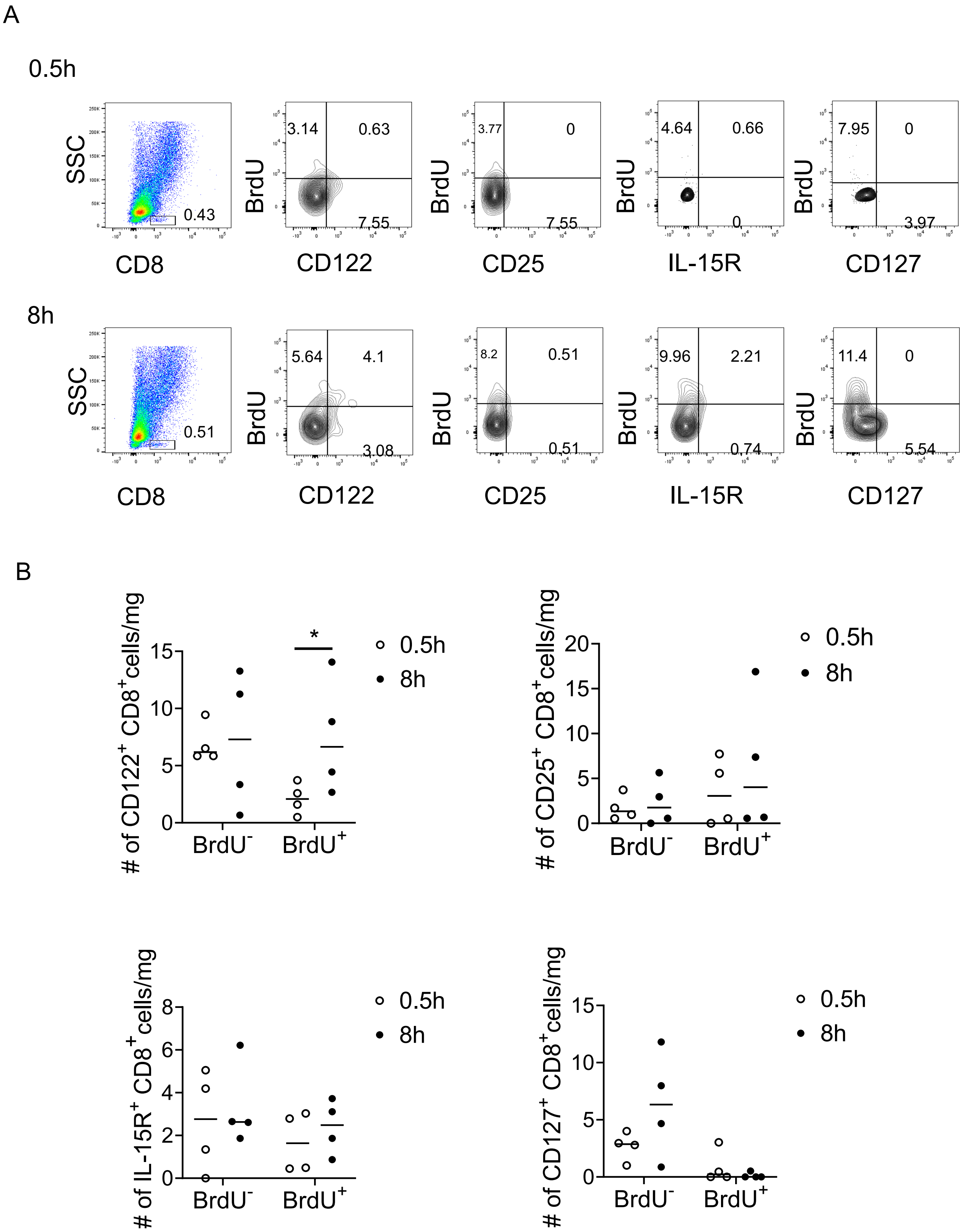

(A) C57BL/6 mice(n=4) were transplanted with A/J cardiac allografts subjected to either 0.5 or 8 hours of CIS. All recipients were injected with 100 μg BrdU i.p. on days 0 and 1 after transplant. On day 2 post-transplant allografts were harvested and digested, and cell suspensions were stained with antibody and analyzed by flow cytometry to assess infiltrating memory $CD8^+$ T cell proliferation and their expression of CD122, CD25, IL-15Ralpha, or CD127. (**B**) Absolute numbers of $CD122^+$, $CD25^+$, IL-$15R^+$ or $CD127^+$ cells in either $BrdU^+$ or $BrdU^-$ $CD8^+$ T cells within the low- or high-ischemic allografts were calculated. *$P < 0.05$ as determined by the Mann-Whitney nonparametric test.

Supplemental Figure 4. Graft-derived dendritic cells are required for extended graft survival in CTLA-4 Ig conditioned recipients of high-ischemic allografts.

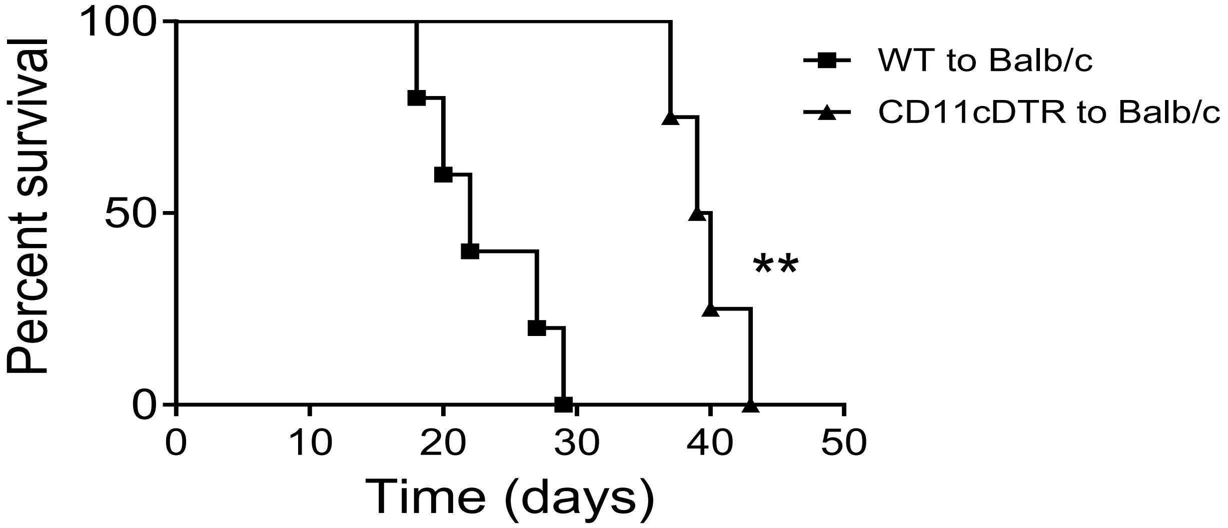

Groups of BALB/c mice (*n* = 4-5/group) received cardiac allografts from C57BL/6 or B6.DTR-CD11c transgenic mice, which were treated with diphtheria toxin (DT; 4 ng/g body weight) on days –2 and –1 prior to transplantation and subjected to 8 hours of cold ischemic storage following donor harvest for transplantation on day 0. All recipients were treated with 200 μg CTLA-4Ig i.p. on days 0 and 1. Graft survival was monitored daily by abdominal palpation and rejection was confirmed visually by laparotomy. **P<0.01 versus survival of C57BL/6 allografts in CTLA-4 treated BALB/c recipients.

Supplement Fig. 5. Expression of inflammatory gene transcripts in allografts subjected to 0.5 or 8 hours cold ischemic storage prior to transplant to recipients treated with or without p40HD

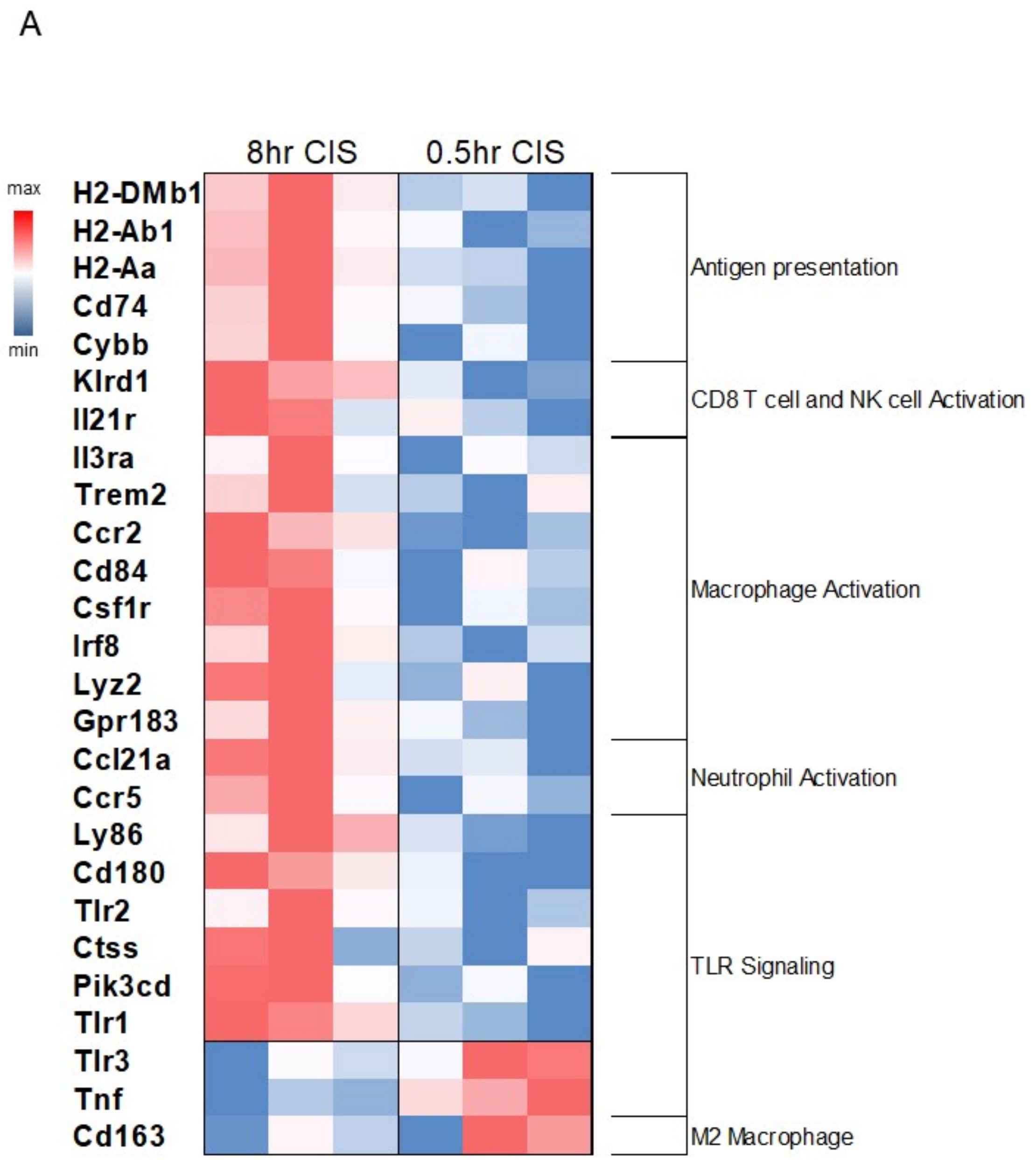

B

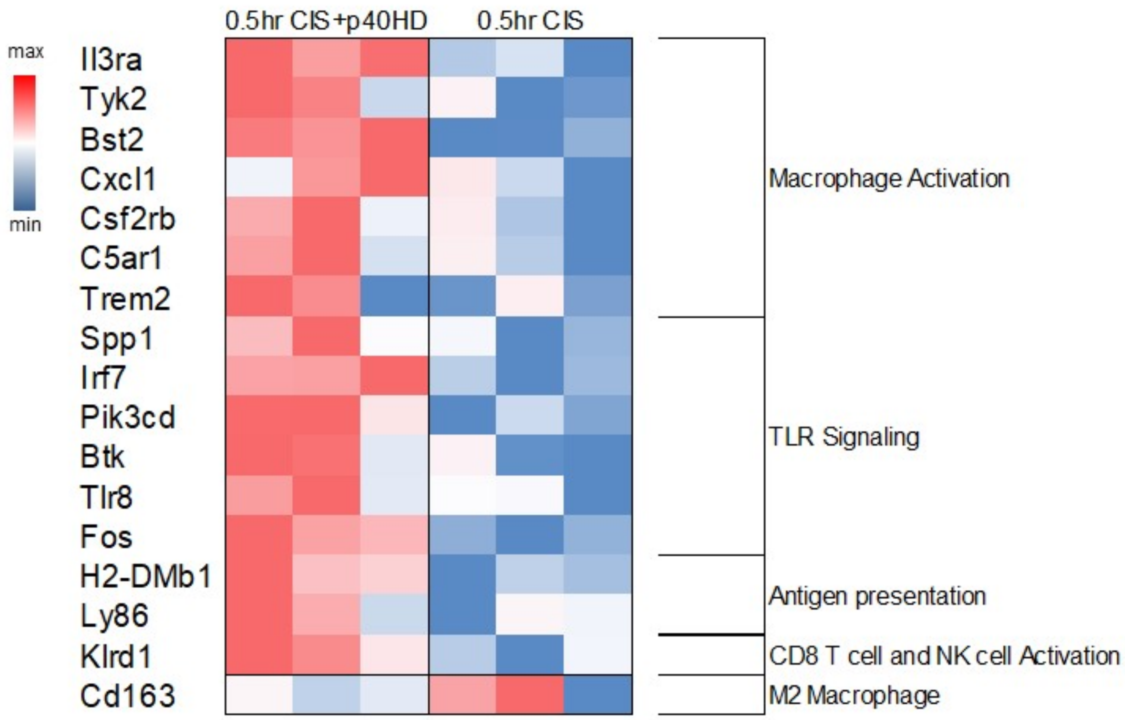
0.5hr CIS+p40HD
0.5hr CIS
max
min
Il3ra
Tyk2
Bst2
Cxcl1
Csf2rb
C5ar1
Trem2
Spp1
Irf7
Pik3cd
Btk
Tlr8
Fos
H2-DMb1
Ly86
Klrd1
Cd163
Macrophage Activation
TLR Signaling
Antigen presentation
CD8 T cell and NK cell Activation
M2 Macrophage

C

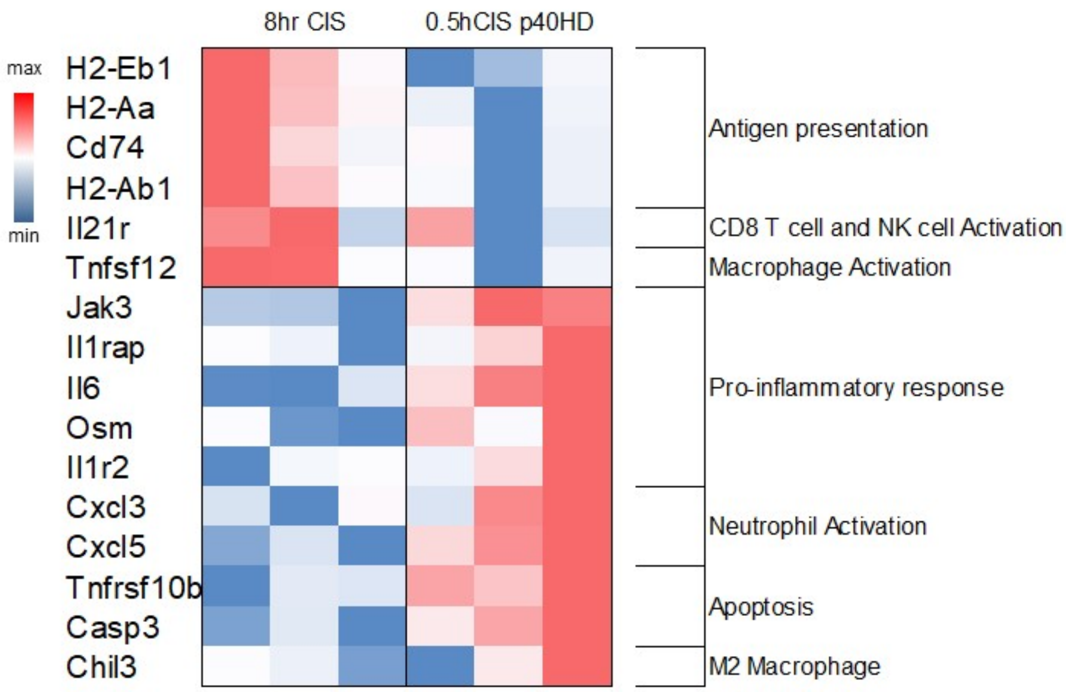
8hr CIS
0.5hCIS p40HD
max
min
H2-Eb1
H2-Aa
Cd74
H2-Ab1
Il21r
Tnfsf12
Jak3
Il1rap
Il6
Osm
Il1r2
Cxcl3
Cxcl5
Tnfrsf10b
Casp3
Chil3
Antigen presentation
CD8 T cell and NK cell Activation
Macrophage Activation
Pro-inflammatory response
Neutrophil Activation
Apoptosis
M2 Macrophage

Groups of C57BL/6 mice (n = 3/group) were transplanted with A/J allografts subjected to either 0.5 or 8 hours of cold ischemic storage (CIS) prior to transplant with the recipients of the 0.5 hour CIS injected with PBS or 2 μg recombinant p40 homodimers i.v on day 1. Grafts were harvested 48 hours after transplant, total RNA was isolated from heart graft homogenates and gene expression was analyzed by the NanoString nCounter platform using the Mouse PanCancer Immune Profiling panel code set. Log_2 normalized counts and expression ratios were generated using nSolver version 4.0 and advanced analysis version 2.0. Heat maps were made from the top differentially expressed genes in Microsoft Excel and shown with red color indicating higher expression than the mean and blue color indicating lower expression than the mean. KEGG analyses of biological pathways were configured from differentially expressed genes in allografts subjected to: (A) 8 vs. 0.5 hours CIS; (B) 0.5 hours CIS in p40 HD treated recipients vs. 0.5 hours CIS in PBS treated recipients; and, (C) 8 hours CIS vs. 0.5 hours CIS in p40 HD treated recipients.

Supplemental Figure 6. Complete differentially express transcript responses of heterologous and donor-primed memory CD8 T cells infiltrating high ischemic cardiac allografts at the time of graft rejection.

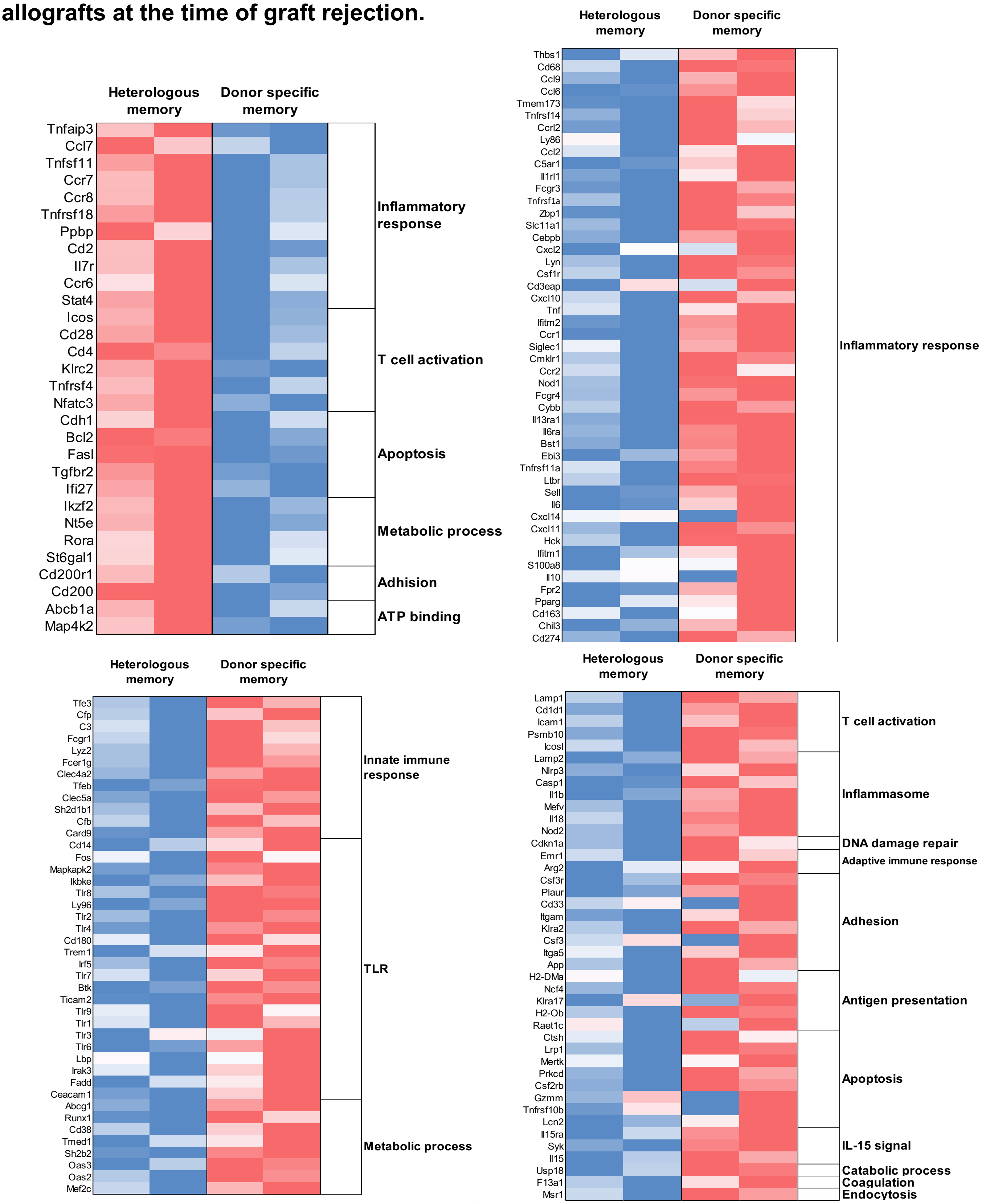

A/J skin allograft primed CD8 T cells from CD45.2+ C57BL/6 recipients were enriched from recipient spleens, 2 x10^6 cell aliquots were transferred to CD45.1+ C57BL/6 mice that were transplanted 3 days later with A/J heart allografts subjected to 8 hours of CIS prior to transplant. CD45.2+ C57BL/6 mice also received high-ischemic A/J allografts. All heart allograft recipients were treated with 250 μg CTLA-4Ig i.p. on days 0 and 1. At the time of graft rejection (day 7 for recipients of donor-primed CD45.2+ CD8 T cells and day 21 for heterologous CD45.2+ memory CD8 T cells), allografts were harvested from the B6.CD45.1+ and CD45.2+ CTLA-4Ig conditioned recipients (n=2-3), were digested to obtain single-cell suspensions, and cell aliquots were stained with fluorochrome-labeled monoclonal antibody to identify the CD44+CD8+ heterologous and transferred donor-primed memory CD8 T cells. The CD8 T cells were purified by flow sorting, RNA was isolated and analyzed by NanoString using the Mouse Pan Cancer Immune Profiling panel. Volcano plots indicate DEG by purified heterologous- versus donor-primed memory CD8 T cells infiltrating A/J allografts. KEGG analyses of biological pathways was derived from DEG of the heterologous versus donor-primed memory CD8 T cells infiltrating allografts with red shaded boxes indicating transcript expression higher than the mean, blue shaded boxes indicate transcript expression lower than the mean.

Supplemental Figure 7: General experimental design of the study

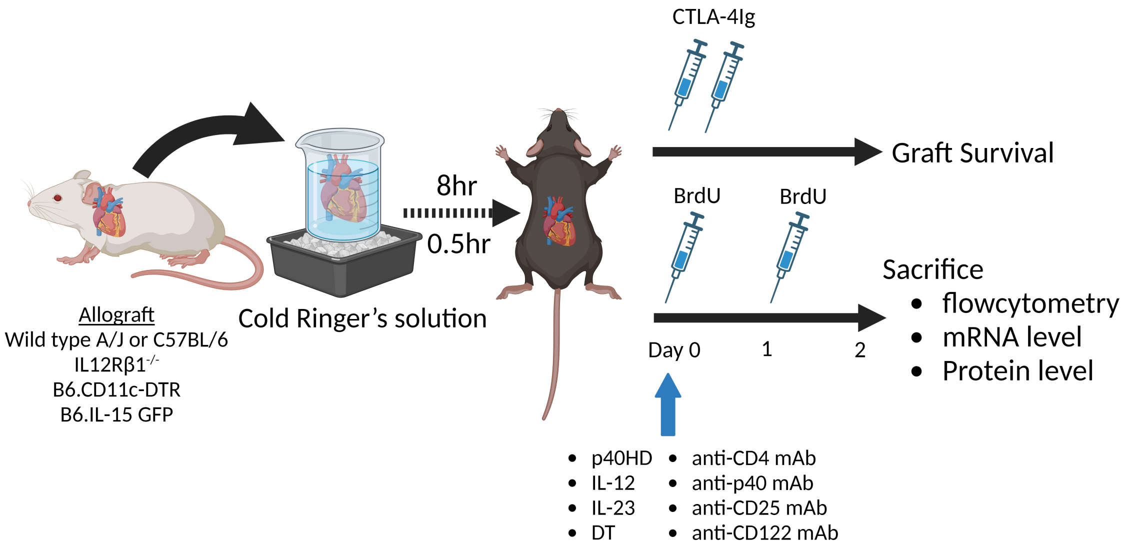

To test mechanisms underlying activation of allograft infiltrating donor-reactive memory CD8 T cells to proliferate within grafts and express functions associated with their CTLA-4Ig resistant rejection of the allografts, allografts and isografts from the indicated donors are harvested and subjected to minimal (0.5 hrs) or prolonged (8 hrs) cold ischemia storage and then transplanted. Groups of the allograft recipients are treated with BrdU on the day of and the day after transplant and with the indicated cytokines or neutralizing antibodies to test their impact on the activation of the memory CD8 T cells to proliferate (by incorporation of BredU) on day 2 post-transplant. Groups of the allograft recipients are also treated with CTLA-4Ig on the day of and the day after transplant and survival of the allografts is monitored by daily palpation.
